# Antimicrobial resistance among agents of community-associated lower respiratory tract infection in the UK and Ireland: trends from 1999/2000 to 2018/2019

**DOI:** 10.1093/jac/dkaf252

**Published:** 2025-10-27

**Authors:** Rosy Reynolds, David Felmingham, Shazad Mushtaq, Carolyne Horner, Aiysha Chaudhry, Rachael Adkin, Michael Allen, Christopher Longshaw, Benjamin J Parcell, David M Livermore

**Affiliations:** Population Health Sciences, University of Bristol, Bristol BS8 2PS, UK; British Society for Antimicrobial Chemotherapy, 53 Regent Place, Birmingham B1 3NJ, UK; Hammerfest, Arrow Lane, Hartley Wintney RG27 8LR, UK; Antimicrobial Resistance and Healthcare Associated Infections Reference Unit, UK Health Security Agency, Colindale, London NW9 5EQ, UK; British Society for Antimicrobial Chemotherapy, 53 Regent Place, Birmingham B1 3NJ, UK; Antimicrobial Resistance and Healthcare Associated Infections Reference Unit, UK Health Security Agency, Colindale, London NW9 5EQ, UK; Antimicrobial Resistance and Healthcare Associated Infections Reference Unit, UK Health Security Agency, Colindale, London NW9 5EQ, UK; British Society for Antimicrobial Chemotherapy, 53 Regent Place, Birmingham B1 3NJ, UK; Medical Affairs, MSD (UK) Limited, 120 Moorgate, London EC2M 6UR, UK; British Society for Antimicrobial Chemotherapy, 53 Regent Place, Birmingham B1 3NJ, UK; Scientific Affairs, Shionogi B.V., Fifty Paddington, 50 Eastbourne Terrace, Paddington W2 6LG, UK; Division of Population Health and Genomics, School of Medicine, University of Dundee, Ninewells Hospital and Medical School, Dundee DD1 9SY, UK; Department of Medical Microbiology, Ninewells Hospital and Medical School, Dundee DD1 9SY, UK; Antimicrobial Resistance and Healthcare Associated Infections Reference Unit, UK Health Security Agency, Colindale, London NW9 5EQ, UK; Norwich Medical School, University of East Anglia, Norwich NR4 7TJ, UK

## Abstract

**Objectives:**

The BSAC Respiratory Surveillance Programme examined resistance trends among *Streptococcus pneumoniae*, *Haemophilus influenzae* and *Moraxella catarrhalis* from patients with community-acquired lower respiratory tract infection (CA-LRTI).

**Methods:**

Quotas of isolates were sought per collecting site from 1999/00 to 2018/19; an annual October start date captured winter infection peaks within single years. MIC testing was by BSAC agar dilution. β-Lactamase detection with nitrocefin and pneumococcal serotyping by classical methods or WGS.

**Results:**

Resistances were uncommon, except that β-lactamases occurred in *c.* 20% of *H. influenzae* from 2012/13 following earlier rises, and in >90% of *M. catarrhalis* throughout. Only 0.11% (12/10881) of *S. pneumoniae* were fully resistant to penicillin; co-amoxiclav inhibited 97.8% of 13526 *H. influenzae* and >99.9% of 6309 *M. catarrhalis* isolates. Cefotaxime inhibited >99% of all isolates at breakpoint, as did relevant fluoroquinolones in the fewer years tested. Tetracycline inhibited >98% of *H. influenzae* and *M. catarrhalis* and 85% of *S. pneumoniae*. Significant shifts were: (i) fluctuating resistances to tetracyclines, macrolides and penicillin in pneumococci, reflecting serotype replacements; (ii) expansion, from 2012/13, in the proportion of *H. influenzae* with β-lactamase-independent amoxicillin/co-amoxiclav resistance; and (iii) increasing high-level amoxicillin resistance (MIC  > 64 mg/L) among β-lactamase-positive *H. influenzae*. MIC differentials were seen for cephalosporins between β-lactamase-positive and β-lactamase-negative *M. catarrhalis*, greatest (512-fold) for ceftaroline.

**Conclusions:**

CA-LRTI remains eminently treatable, yet shifts are occurring in the serotypes of *S. pneumoniae* most associated with resistance and in the nature of amoxicillin resistance in *H. influenzae*. β-Lactamase-related cephalosporin MIC differentials for *M. catarrhalis* are striking but their clinical significance remains uncertain.

## Introduction

Acute community-acquired lower respiratory infections (CA-LRTI), including community-acquired bacterial pneumonia (CAP), are frequent, increasing with age and being more frequent in the north than the south of England.^1^ Rates are higher in men than women and among those with socioeconomic deprivation, chronic underlying respiratory disease or a history of smoking and immunosuppression.^1^ The associated mortality is 5%–15% among those hospitalized with bacterial CAP, rising to 30% among those admitted to ICU.^2^ There was a remarkable suppression of bacterial CAP, as well as invasive pneumococcal disease, during the COVID-19 pandemic lockdowns of 2020–21, perhaps because transmission was prevented, or because the seasonal viral infections that often precipitate bacterial pneumonia were suppressed.^3,4^

The ‘typical’ pathogens of CA-LRTI are *Streptococcus pneumoniae*, *Haemophilus influenzae* and, less frequently, *Moraxella catarrhalis*,^5^ although laboratory failure to recover a pathogen is frequent^6^ and may reflect sample quality, the challenges of culturing delicate and fastidious organisms or a larger-than-recognized proportion of viral pneumonias. A minority of cases, concentrated among younger patients, involve ‘atypical’ pathogens, notably *Mycoplasma pneumoniae* and *Chlamydophila pneumoniae*.^5^  *Legionella pneumophila*, another ‘atypical’ agent, notoriously causes outbreaks when aerosolized from air-conditioning or other water-containing systems.^7^ Severe disease is most often associated with *S. pneumoniae* or *L. pneumophila*, although the latter is uncommon.^5,7^

The BSAC resistance surveillance monitored resistance trends in the ‘typical’ pathogens of CA-LRTI from October 1999 to September 2019, and results are presented here. This period included the years—2006 and 2010, respectively—when 7- and 13-valent pneumococcal conjugate vaccines (PCVs) were first deployed in UK children as protection against invasive pneumococcal disease.^8^ It is now well established that, via a herd-immunity effect, these also reduce invasive pneumococcal disease in older cohorts.^9^ Reductions in vaccine-serotype non-invasive pneumonia have also been reported, although less extensively.^10^

The impacts of vaccines on serotype distributions in both bacteraemia and CA-LRTI are discussed more fully elsewhere in this Supplement, as are the effects on particular serotype-associated resistances.^11^ This paper describes the resistance changes in pneumococci from CA-LRTI during the two decades of the BSAC’s surveillance. It also describes changing resistance patterns in *H. influenzae* and raised MICs for cephalosporins—notably anti-MRSA ‘fifth-generation’ agents—in β-lactamase-producing *M. catarrhalis*.

## Materials and methods

Details of methods are fully described elsewhere in this supplement^12^; accordingly, only a brief summary is provided here. Isolates were requested from patients with CA-LRTI in community settings or hospitalized for ≤48 h. They were collected over ‘winter’ seasons (defined as October to April) from 1999/2000 until 2007/08 and in rolling October to September years thereafter until 2018/19; 34–39 laboratories contributed each season in 2010/11 to 2014/15, and 20–25 laboratories in all other seasons. The surveillance sought up to 1000 isolates each of *S. pneumoniae* and *H. influenzae* and 500 *M. catarrhalis* until 2007/08, with these approximately halved from 2008/09 to 500–560 and 250–280, respectively. Identification was originally by classical methods, later replaced by MALDI-TOF for *H. influenzae* and *M. catarrhalis*. MICs were determined by BSAC agar dilution and categorized for susceptibility and resistance against EUCAST 2022 breakpoints; β-lactamase detection was with nitrocefin; pneumococcal serotyping initially followed classical methods but was later inferred from WGS. The antibiotics tested included core agents tested in all years under the aegis of the BSAC as well as those included for variable periods contingent on sponsorship by funders. All these aspects are generic to the data papers of this supplement and are fully described elsewhere.^12^

Tables S1 and S2 (available as Supplementary data at *JAC* Online) show the numbers of laboratories contributing, isolates collected per season, and necessary data interpretations and exclusions. Tables S3–S5 detail breakpoints (EUCAST v12.0, 2022) and susceptibility tests by organism, antimicrobial and years included. Tables S6–S9 and Figures S1 and S2 cover patient characteristics, noting any missing data. MIC distributions are presented as an Appendix to the Supplementary Data.

### Analysis

Analysis was descriptive and largely graphical, using Stata 18.0 (StataCorp LLC: College Station, TX, USA) and Bischoff’s colour vision-sensitive ‘plotplainblind’ graph scheme.^13^ Missing data were excluded in the calculation of percentages.

## Results

### Isolate collection

The total collection comprised 10 881 isolates of *S. pneumoniae*, 13 526 *H. influenzae* and 6309 *M. catarrhalis* (Table S2). Greater numbers of *H. influenzae* than *S. pneumoniae* may reflect greater prevalence, or better pathogen recovery, given that target numbers of isolates were identical for both species. For all three species, over 90% of isolates were from sputum specimens, with small minorities from other sources, most frequently broncho-alveolar lavage (Table S9). The proportion of male patients averaged between 49% (*M. catarrhalis*) and 55% (*S. pneumoniae*), with some indication of a downward trend from near 60% to around 50% over the surveillance period for *S. pneumoniae* (Table S6). The median patient age ranged from 63 (*S. pneumoniae*) to 67 (*M. catarrhalis*) years; the proportion of patients aged ≥80 years was higher for *M. catarrhalis* (16%) than for *S. pneumoniae* (12%) and *H. influenzae* (11%) (Table S7 and Figure S1). *M. catarrhalis* has also been associated with older patients in Japan.^14^ Patients aged under 1 year formed a distinct but small group, largest for *M. catarrhalis* at 2%. The reported proportion of hospitalized patients (≤48 h) fell from a peak of near 60% in 2003/04 to stabilize near 10% from about 2014/15 onwards for all three species (see Figure S2); we suspect that this may partly reflect how specimens from Admissions Unit and Accident and Emergency are recorded in hospital data systems, as well as increased care in the community, including Outpatient Parenteral Antimicrobial Therapy.^15^

### S. pneumoniae

**Figure 1.**
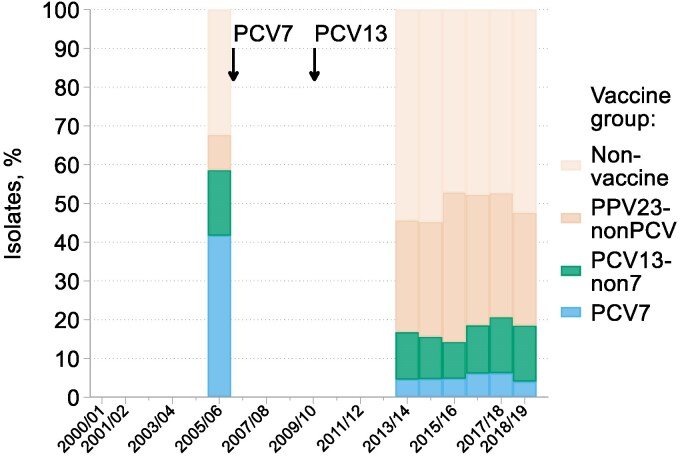
Changing prevalence of vaccine serotypes among *S. pneumoniae* from CA-LRTI. Arrows indicate when PCV7 and PCV13 were introduced to the infant vaccination schedule in the UK; wide deployment of PCV7 followed a year later in Ireland whereas PCV13 deployment was concurrent in the UK and Ireland. Serotypes included in each vaccine group were PCV7: 4, 6B, 9V, 14, 18C, 19F and 23F; PCV13-non7: 1, 3, 5, 6A, 7F and 19A; PPV23-nonPCV: 2, 8, 9N, 10A, 11A, 12F, 15B, 17F, 20, 22F and 33F; and non-vaccine: any serotype not included in PCV7, PCV13 or PPV23.

The collection of *S. pneumoniae* comprised 661–809 isolates per season from 1999/2000 to 2007/08 then, with altered quotas and testing at a different central laboratory, 325–480 isolates per season from 2008/09 to 2018/19 (Tables S2 and S3). Isolates were serotyped in 2005/06 and then continuously from 2013/14 to 2018/19. Proportions within the coverage of different pneumococcal vaccines changed substantially between these periods (Figure 1); more details are provided elsewhere in this Supplement.^11^

**Figure 2.**
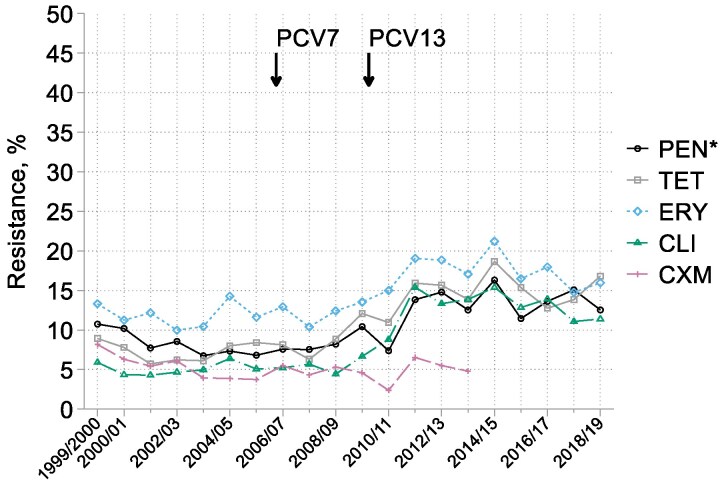
Resistance trends among *S. pneumoniae* from CA-LRTI. PEN*, penicillin (with ‘susceptible, increased exposure’ included as resistant); TET, tetracycline; ERY, erythromycin; CLI, clindamycin; CXM, cefuroxime. Arrows indicate when PCV7 and PCV13 were introduced to the infant vaccination schedule in England.

After seven seasons of stability (Figure 2), the period from 2007/08 to 2012/13 witnessed an approximate doubling in the prevalence of resistance among pneumococci to erythromycin (10%–19%), clindamycin (6%–13%) and tetracycline (6%–16%) and of reduced susceptibility to penicillin (MIC  > 0.06 mg/L; 8%–15%); inducible clindamycin resistance, tested from 2011/12, was very rare (0.4%), accounting a tiny fraction of all clindamycin resistance. Subsequently, from 2014/15, there were reductions in resistance or reduced susceptibility to all these agents; however, these falls were smaller than the preceding rises and, for penicillin and tetracycline, were short-lived. Underlying these rather sedate changes were much bigger serotype shifts, doubtless contingent on PCV deployment. In 2005/06, most resistance to erythromycin and tetracyclines and non-susceptibility to penicillin was associated with isolates of Serotypes 6B, 9V, 14, 15, 19F and 23F (Table 1). By 2013/14 to 2015/16, resistance or reduced susceptibility to all these agents was largely associated with isolates of Serotypes 15A (especially), 3, 19A, 19F, 23B, 33F and 35B. These serotypes remained the most prominent among resistant isolates in 2016/17 to 2018/19, as explored more fully elsewhere in this Supplement.^11^ Notably, the proportion of clindamycin resistance among erythromycin-resistant isolates increased from 44% in 2005/06 to 77% and 75% in 2013/14 to 2015/16 and 2016/17 to 2018/19, respectively.

**Table 1.** Proportions of CA-LRTI *S. pneumoniae* isolates with resistance/reduced susceptibility to penicillin, erythromycin and tetracycline, and top four serotypes of resistance burden, before and after PCV introduction

Period	2005/06	2013/14 to 2015/16	2016/17 to 2018/19
Total isolates of *S. pneumoniae* from CA-LRTI	*N* = 749	*N* = 1162	*N* = 1021
Penicillin I^a^ + R^b^ (MIC > 0.06 mg/L)			
*N* (%) penicillin I + R	51 (6.8%)	158 (13.6%)	140 (13.7%)
*N* (%) of penicillin I + R due to PCV13 types	45 (88%)	45 (28%)	36 (26%)
Top four types for penicillin I + R (rank order)	14 9V 19F 6B	15A 19A 35B 19F	15A 23B 19F 35B
% penicillin I + R due to top four types	75%	63%	56%
Erythromycin R^b^ (MIC > 0.5 mg/L)			
*N* (%) erythromycin R	87 (11.6%)	214 (18.4%)	166 (16.3%)
% of erythromycin R due to PCV13 types	65 (75%)	54 (25%)	49 (30%)
Top four types for erythromycin R (rank order)^c^	14 19F 6B 15^c^	15A 19F 33F 19A	15A 19F 19A 35B
% of erythromycin R due to top four types	70%	55%	51%
Clindamycin R^b^ (MIC > 0.5 mg/L)			
*N* (%) clindamycin R	38 (5.1%)	164 (14.1%)	124 (12.1%)
% of clindamycin R due to PCV13 types	31 (82%)	50 (30%)	42 (34%)
Top four types for clindamycin R (rank order)	19F 14 6B 15	15A 19F 33F 19A	15A 19F 19A 33F
% of clindamycin R due to top four types	82%	68%	60%
Tetracycline *R*^b^ (MIC > 2 mg/L)			
*N* (%) tetracycline R	63 (8.4%)	187 (16.1%)	148 (14.5%)
% of tetracycline R due to PCV13 types	44 (70%)	76 (36%)	67 (45%)
Top four types for tetracycline R (rank order)^c^	19F 6B 14 23F^c^	15A 19F 3 19A	15A 3 19F 19A
% of tetracycline R due to top four types	62%	59%	63%

^a^I = susceptible (increased exposure).

^b^R = resistant.

^c^Isolates that could not be assigned to a serogroup ranked fourth among erythromycin-R and tetracycline-R in 2005/06, ahead of Serotypes 15 and 23F, respectively, but are demoted as they were heterogeneous.

Only 12 *S. pneumoniae* isolates out of all 10881 were fully resistant to penicillin, with MICs > 2 mg/L; nine of these 12 were from Ireland, as against 1458/10 881 (13%) of all isolates; seven were from a single centre that accounted for half of the Irish isolates and were scattered across years. Penicillin MICs were either 4 (*N* = 11) or 8 mg/L (*N* = 1); only two resistant isolates were serotyped, and both belonged to type 19F. All other penicillin non-susceptibility was low level, with MICs 0.12–2 mg/L and most often (64%) in the lower part of that range, with values of 0.12–0.5 mg/L. Amoxicillin resistance, at the breakpoint for oral administration (*R*  > 1 mg/L), increased from an average of 1% in the first five annual collection periods to 3% in the last five; cefotaxime resistance remained <1% throughout. Cefuroxime resistance, tested until 2013/14, was fairly stable at around 5%. Resistance rates were under 1% for four other β-lactams and fluoroquinolones that were not tested in all years, specifically ceftaroline (mode, 0.008; range, 0.002–0.5 mg/L); ceftobiprole (0.015; 0.004–2 mg/L); levofloxacin (1; 0.25–64 mg/L); and moxifloxacin (0.12; 0.03 to ≥64 mg/L). Twelve percent of the pneumococci were resistant to cefaclor (mode 0.25; range 0.015 to ≥256 mg/L), a drug tested only until 2004/05. Clarithromycin was tested for 4 years, giving results that paralleled those for erythromycin (Table S3).

MICs of ceftaroline and ceftobiprole correlated strongly, but ceftaroline was slightly more active based on MICs, and with fewer resistant isolates (one versus 11) among 1021 tested with both agents; a fuller comparison was published in 2020.^16^

### H. influenzae

**Figure 3.**
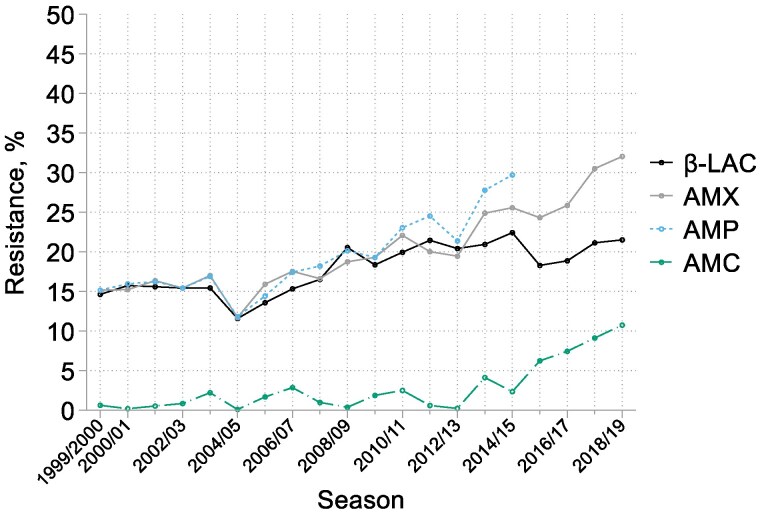
Trends in resistance and β-lactamase production among *H. influenzae* from CA-LRTI. β-LAC, β-lactamase; AMX, amoxicillin; AMP, ampicillin; AMC, co-amoxiclav. N.B. Co-amoxiclav was tested with a 2:1 amoxicillin:clavulanate ratio until 2012/13 and with a fixed 2 mg/L clavulanate from 2013/14. This shift, increasing the effective breakpoint from >2 + 1 to >2 + 2 mg/L, should have slightly suppressed rather than increased resistance rates.

**Figure 4.**
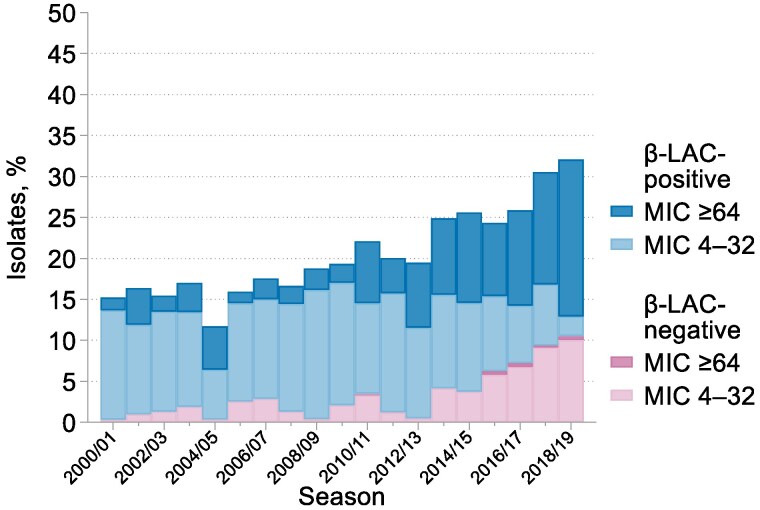
Levels of amoxicillin resistance among amoxicillin-resistant CA-LRTI *H. influenzae* with or without β-lactamase activity. β-LAC, β-lactamase; MIC, mg/L of amoxicillin. Results for 1999/2000 are excluded as MIC tests were censored at 16 mg/L.

**Figure 5.**
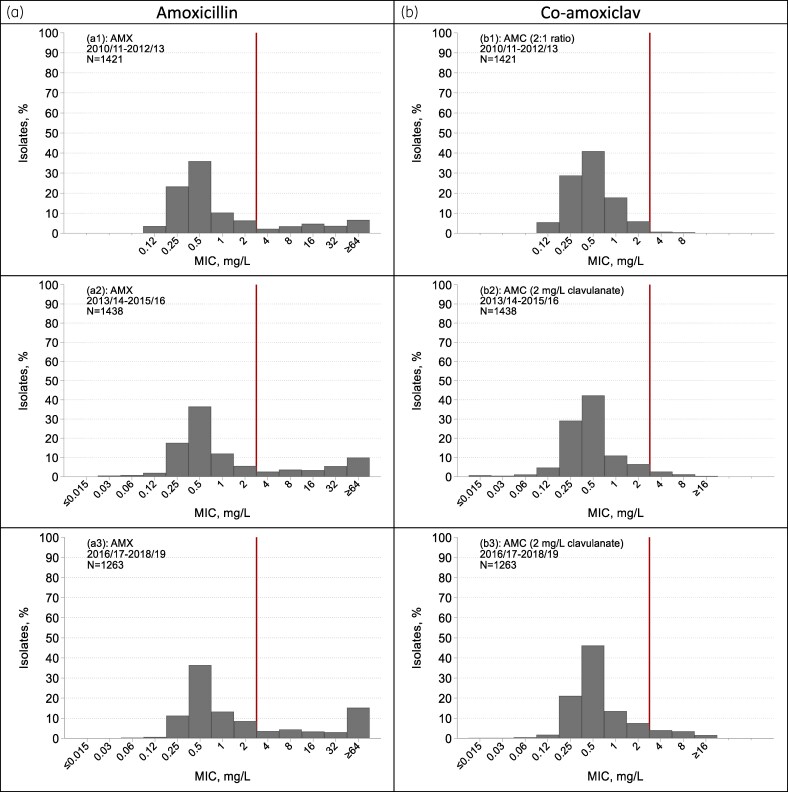
MIC distributions for (a) amoxicillin (AMX) and (b) co-amoxiclav (AMC) among *H. influenzae* from CA-LRTI for three 3-year periods—(top panel) before, (middle panel) after and (bottom panel) longer after the change of central testing laboratory and testing format for co-amoxiclav (which occurred between 2012/13 and 2013/14). In the first period (2010/11 to 2012/13, top panels), testing was at LGC and co-amoxiclav testing was with a 2:1 amoxicillin:clavulanate ratio. In both later periods (2013/14 to 2015/16, middle panel; also 2016/17 to 2018/19, bottom panel), testing was at UKHSA and co-amoxiclav testing was with a fixed 2 mg/L clavulanate. There was no upward shift of modal or lower MICs in the 3 years after the change, nor subsequently. Rather, the proportion of isolates with resistance increased over time while the laboratory and method remained constant (see Figures 3 and 4). The MIC axis spans ≤0.015 to ≥64 mg/L in all cases, with labelled values showing the range of observed data; the red vertical line indicates the resistance breakpoint.

The 13 526 *H. influenzae* comprised 888–1004 isolates in each of the first nine seasons and 416–528 in those from 2008/09 onwards (Tables S2 and S4). The prevalence of β-lactamase production (as detected with nitrocefin) and resistance to both amoxicillin and ampicillin (as estimated by MIC determinations) were stable and near equal at around 16% in the five seasons to 2003/04, before increasing gradually to near 20% in 2012/13. After 2012/13, their paths diverged: β-lactamase production remained near 20%, whereas total amoxicillin resistance continued to rise, reaching 32% in 2018/19 (Figure 3). This divergence reflected the expansion of β-lactamase-negative amoxicillin-resistant (BLNAR) organisms, from fewer than 2% of isolates in 1999/2000 to 2012/13 to 11% by 2018/19 (Figure 4). Resistance to co-amoxiclav climbed in parallel with the rise of the BLNAR isolates over the same period and from a similarly low baseline (Figure 3). BLNAR isolates typically had low-level amoxicillin resistance, with MICs of 4 or 8 mg/L in 84% of cases; amoxicillin MICs for β-lactamase producers mostly were higher. Since the modal MICs of amoxicillin and co-amoxiclav for susceptible isolates remained constant over time at 0.5 mg/L, we consider that these shifts were real and not artefacts of testing nor the change of central testing laboratory (Figure 5).

Also notable, and beginning around 2009/10 (i.e. slightly before the rise of BLNAR), there was a striking increase in the proportion of β-lactamase producers with high-level amoxicillin resistance, defined here as an amoxicillin MIC  ≥ 64 mg/L. From 2000/01 to 2009/10, this trait was seen, on average, for 18% of β-lactamase producers and 3% of all *H. influenzae* isolates, increasing to 89% and 19% respectively by 2018/19 (Figure 4). Most β-lactamase producers [96% on average but only 90% (72/80) in the final surveillance year] remained susceptible to co-amoxiclav despite the high amoxicillin MICs; overall, the mode MIC of co-amoxiclav for β-lactamase producers was 0.5 mg/L, with a range from 0.03 to 8 mg/L.

*H. influenzae* had low rates of resistance to other agents that were tested every season, at <1% for cefotaxime (MIC mode 0.015 mg/L; range ≤0.001 to 1 mg/L) and ciprofloxacin (0.008; ≤0.001 to ≥64 mg/L) and <2% for tetracycline (0.5; ≤0.03 to ≥16 mg/L) (see MIC distributions in Appendix to Supplementary Data). Resistance to cefuroxime, which was tested only in the first 15 years, averaged 6% (MIC mode 0.05 mg/L; range ≤0.03 to ≥64 mg/L); it was seen for 5% of co-amoxiclav-susceptible isolates but for 66% of co-amoxiclav-resistant isolates. Unfortunately, testing of cefuroxime was terminated in 2013/14, at around the date when co-amoxiclav resistance began to rise notably, precluding later cross-correlation of these traits.

Based on three to nine years of testing, resistance rates were <1% for moxifloxacin (mode 0.03; range 0.004–8 mg/L), levofloxacin (0.015; 0.004–16 mg/L) and ertapenem (0.03; 0.004–1 mg/L); 2% for minocycline (0.25; 0.03–8 mg/L); and 3% for ceftaroline (0.008; ≤0.002 to 1 mg/L). MIC modes and ranges for four agents lacking breakpoints, but tested in 6–16 seasons, were: cefaclor (2; 0.12 to ≥256 mg/L), ceftobiprole (0.06; ≤0.002 to 2 mg/L), tigecycline (0.25; 0.03 to 2 mg/L) and trimethoprim (0.12; ≤0.015 to ≥512 mg/L).

EUCAST has no breakpoints for macrolides against *H. influenzae*, citing conflicting evidence in respect of efficacy, but does indicate ECOFFs of 16 mg/L for erythromycin and 32 mg/L for clarithromycin. We found MICs exceeding these values for <2% of isolates in the case of erythromycin (mode MIC, 4; range ≤0.03 to ≥512 mg/L), which was tested every year until 2014/15, and <1% for clarithromycin (mode, 4; range, 0.06 to ≥128 mg/L), which was tested only from 1999/2000 to 2002/03.

### M. catarrhalis

A total of 6309 *M. catarrhalis* isolates were collected: 403–461 per season from 1999/2000 to 2007/08 and 190–270 in each of the following 11 seasons. β-Lactamases were detected, using nitrocefin, in 94% of these isolates (see Tables S2 and S5). Fluoroquinolone resistance, detected using nalidixic acid as a screen, was seen in fewer than 1% of isolates, with no notable trends over time. MICs of all antibiotics were determined at high inoculum (*c.* 10^6^/spot), in accordance with BSAC and EUCAST guidance.^17^

Co-amoxiclav, ciprofloxacin, erythromycin and tetracycline MICs were determined in 18 seasons (omitting 2001/02 and 2003/04 for financial reasons). All these antibiotics had resistance rates below 1%, without trends. MIC modes and ranges were co-amoxiclav (0.12; ≤0.001 to 0.5 mg/L), ciprofloxacin (0.03; 0.008–0.03 mg/L), erythromycin (0.06; 0.008–2 mg/L) and tetracycline (0.5; 0.06 to ≥16 mg/L) (see MIC distributions in Appendix to Supplementary Data). Based on fewer (8–12) seasons’ data, minimal resistance rates also were seen for cefotaxime (mode 0.5; range 0.015–2 mg/L), cefuroxime (1; 0.06–16 mg/L) and minocycline (0.12; 0.015–0.5 mg/L) and over 3–4 seasons for clarithromycin, levofloxacin and moxifloxacin. MIC modes and ranges for seven agents lacking breakpoints, tested in 3–9 seasons, were amoxicillin (16; 0.002 to ≥512 mg/L), ampicillin (16; 0.002 to ≥512 mg/L), cefaclor (4; 0.03–128 mg/L), ceftaroline (4; ≤0.002 to ≥8 mg/L), ceftobiprole (1; 0.008 to ≥8 mg/L), tigecycline (0.12; 0.03–0.5 mg/L) and trimethoprim (16; 1 to ≥1024 mg/L).

**Figure 6.**
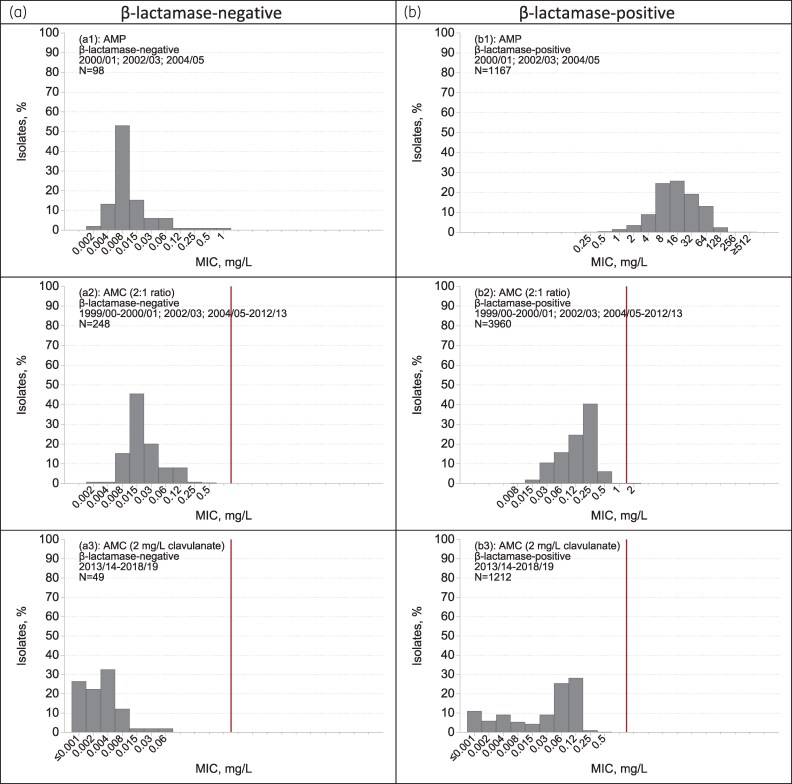

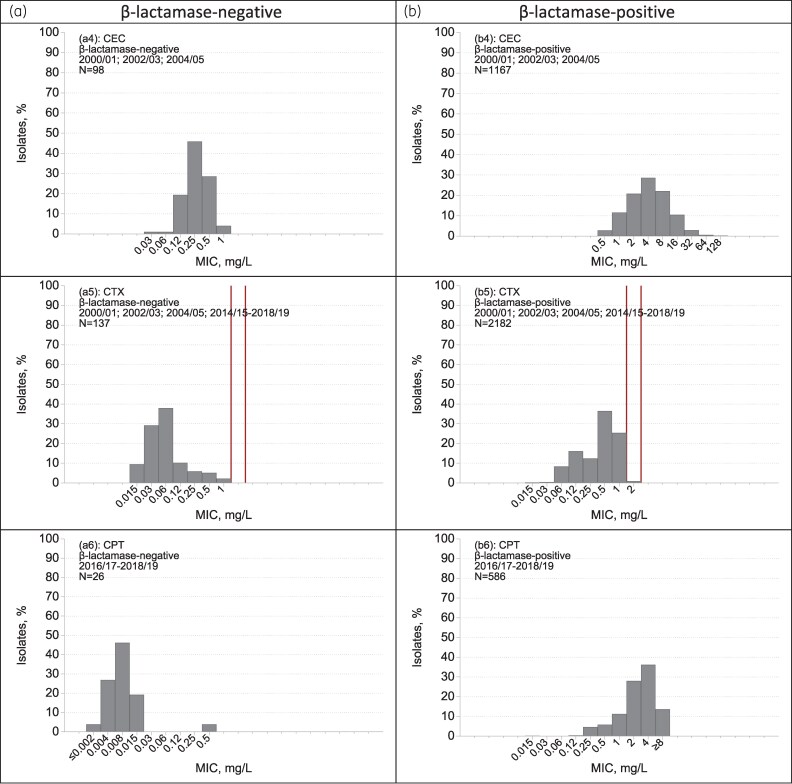

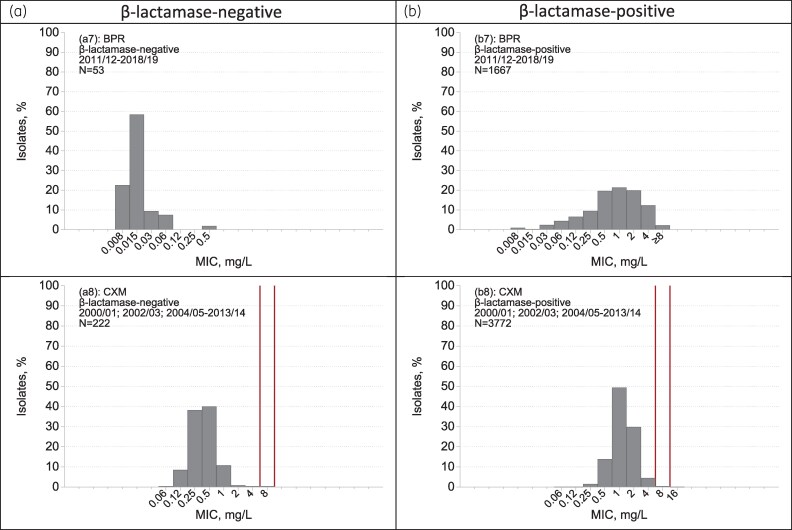
MIC distributions for β-lactamase-negative (a) and β-lactamase-positive (b) *M. catarrhalis* from CA-LRTI. AMP, ampicillin (1999/2000 omitted); AMC, co-amoxiclav; CEC, cefaclor; CTX, cefotaxime; CPT, ceftaroline; BPR, ceftobiprole; CXM, cefuroxime. Years with data affected by testing of a restricted concentration range were omitted, as noted, to show the true ranges more accurately. The MIC axis spans ≤0.001 to ≥1024 mg/L in all cases, with labelled values showing the range of observed data; red vertical line(s) show breakpoints. Where two lines are shown, they indicate the susceptible (S ≤) and resistant (R >) breakpoints; MICs between these bounds are designated I ‘susceptible, increased exposure’. Figure continues on next two pages.

Despite the high susceptibility rates for β-lactams other than ampicillin, strong β-lactamase-related MIC effects were seen for many compounds, as illustrated in Figure 6. For cefuroxime, the ratio of modal MICs for β-lactamase-producing versus non-producing isolates was 2-fold (1 versus 0.5 mg/L) compared with 8-fold for cefotaxime (0.5 versus 0.06 mg/L), 16-fold for cefaclor (4 versus 0.25 mg/L), 32-fold for co-amoxiclav tested at 2:1 ratio (0.5 versus 0.015 mg/L), 64-fold for ceftobiprole (1 versus 0.015 mg/L) and 512-fold for ceftaroline (4 versus 0.008 mg/L). Co-amoxiclav, as tested with a fixed 2 mg/L clavulanate from 2015/16 to 2018/19, is omitted from this list and Figure 6 because, at 2 mg/L, clavulanate alone inhibited 7% of *M. catarrhalis* isolates.

## Discussion

There is little shortage of effective agents against the typical agents of CA-LRTI in the UK and Ireland. Co-amoxiclav retained activity against all except 1/5469 (>99.9%) *M. catarrhalis* tested and against 13 228/13 526 (97.8%) of *H. influenzae*, although against only 1148/1263 (90.9%) in the final 3 years, reflecting the recent expansion of BLNAR isolates. Amoxicillin was active against 98.4% of *S. pneumoniae* overall and 96.4% in the final 3 years, with only 12/10 881 showing substantive penicillin resistance. Cefotaxime retained activity against 99.5% of the 13 526 *H. influenzae* collected, against all 2319 *M. catarrhalis* isolates tested during the eight seasons that it was included, and against 10 875/10 881 (99.9%) pneumococci. Levofloxacin and moxifloxacin were only tested in a few of the surveillance years, reflecting their limited adoption for CA-LRTI in the UK; importantly, though, both these fluoroquinolones retained activity against over 99% of isolates of each of the three species whenever they were tested including, most recently, in 2015/16 (moxifloxacin) and 2018/19 (levofloxacin). Tetracycline retained activity against over 98% of isolates of the two Gram-negative pathogens, with significant resistance (14.5% averaged over the final three surveillance years) only in *S. pneumoniae*. Among newer agents, both ceftaroline and ceftobiprole had excellent activity against *S. pneumoniae*, with MICs related to those of cefotaxime but slightly lower (see also Horner *et al*.^16^). Both also had universally low MICs for *H. influenzae* isolates.

Despite this reassuring picture, there are undercurrents of concern in respect of all three species: serotype-associated shifts for *S. pneumoniae*; rising proportions of BLNAR and highly-ampicillin-resistant β-lactamase producers in the case of *H. influenzae*, and the large MIC differentials between β-lactamase producers and non-producers for newer cephalosporins in the case of *M. catarrhalis*.

In the case of *S. pneumoniae*, the apparent slow uptrends in resistance to macrolides, tetracyclines and reduced susceptibility to penicillin illustrated in Figure 2 disguise a substantial switch in the serotypes involved—as discussed more fully elsewhere in this Supplement.^11^ In 2006/07—the sole pre-2013/14 year when serotyping was undertaken—most antibiotic-resistant pneumococci belonged to Serotypes 6B, 9V, 14, 19F and 23F. These all lie within the spectra of PCV7 and PCV13, which were deployed in children in 2006 and 2010, respectively.^9^ Among these five vaccine-covered types, only 19F remained prominent from 2013/14 onwards, when serotyping of respiratory pneumococci became part of the agreed BSAC CA-LRTI protocol. Subsequently, continuing to 2018/19, the types most associated with resistance to macrolides and tetracyclines and reduced susceptibility to penicillin were Serotypes15A (especially), 23B, 33F and 35B, which are covered by neither PCV7 nor 13, along with serotype 3 and 19A, which are within the spectrum of PCV13, but not PCV7.

Owing to these shifts, the percentage resistance plots on Figure 2 should be read not as straightforward trends but, rather, as the overlapping of two of more resistance peaks, the earlier one composed of PCV7 types and the later comprising non-vaccine types, particularly 15A and 35B, along with those PCV7/13 types that have either evaded PCVs to some degree or have been suppressed less effectively in pneumonia than in invasive infections, notably serotypes 3, 19A and 19F.^18^ Overall, the prevalence rates of resistance to macrolides, tetracyclines and reduced susceptibility to penicillin are now higher in CA-LRTI than among invasive isolates, as discussed elsewhere,^19^ reflecting the fact that serotype 15A, often multiresistant, has become one of the most prevalent serotypes in CA-LRTI, whereas invasive infections now substantially involve rarely-resistant serotypes, notably 8 (especially), 12F and 22F.^11^ Also of note, the proportion of macrolide-resistant pneumococci from CA-LRTI that were resistant to clindamycin rose markedly, from 44% in 2005/06 to 76% in 2013/15 to 2018/19. This likely reflects an increasing proportion of macrolide resistance in recent years being attributable to *erm* genes, encoding methylases that modify the ribosome to block binding of both macrolides and clindamycin rather than *mef* genes, which encode macrolide-specific efflux pumps that do not recognize lincosamides.^20^ Direct evidence for this assertion is that *erm*(B) genes are prevalent in multiresistant serotype 15A^21^ whereas the *mef* genes cause most macrolide resistance in serotype 14,^22,23^ which was the most frequent serotype for macrolide resistance in the 2006/07 ‘snapshot’. It is notable that most of the few fully penicillin-resistant pneumococci collected (9/12) came from Ireland: EARS-net data consistently show higher rates of this resistance among bloodstream pneumococci in Ireland than the UK.^24^ However, the BSAC surveillance had too few sites in Ireland to support robust analysis of this aspect.

Turning to *H. influenzae*, two important shifts are evident: first, the rise in BLNAR, affecting susceptibility rates for both amoxicillin and co-amoxiclav from around 2013 and, second, from slightly earlier, an expansion of highly amoxicillin-resistant β-lactamase-producing isolates with MICs  ≥ 64 mg/L. It might be suspected that these shifts reflected the near-concurrent (2013/14) transfer of the LRTI Programme from LGC to PHE (now UKHSA), but this interpretation seems unlikely for two reasons. First, because, while a change in central laboratory might lead to a step change in the proportion of isolates scored as borderline resistant, it cannot reasonably account for a rising resistance trend across multiple subsequent years and, second, because modal MICs for multiple antibiotics, including amoxicillin and co-amoxiclav, did not change between the pre- and post-move periods (Figure 5). Accordingly, we believe the identified trends to be genuine.

Without genotyping, it is impossible to say whether these resistance shifts in *H. influenzae* reflect repeated emergence of new resistance or clonal expansion. BLNAR substantially owe their resistance to changes in PBP3,^25^ sometimes augmented by up-regulated efflux.^26,27^ Clonal spread of strains with these mechanisms has been reported in Japan,^28^ Norway,^29^ Sweden^30^ and Spain^31^ whereas polyclonal expansion has been recorded in Japan^32^ and Czechia,^33^ with patterns complicated by potential strain-to-strain transformation of *ftsI*, encoding the modified PBP3.^34^ In the case of rising high-level amoxicillin resistance among β-lactamase producers, the likely mechanism is increased β-lactamase production, given the continued full susceptibility to co-amoxiclav, which would be compromised by PBP or reduced uptake.

In the case of *M. catarrhalis*, 94% of isolates were found to produce β-lactamase, compared with 90.7% in a multicentre UK survey performed in 1991.^35^ The modal MIC of ampicillin in the BSAC surveillance was 8–16 mg/L. This is 4- to 8-fold higher than the geometric mean MIC of 2 mg/L, also determined with a 10^6^ cfu/spot inoculum, found by Yeo and Livermore^36^ for isolates from the 1991 collection with BRO-1, which is considerably the predominant β-lactamase in the species. A geometric mean MIC of 16 mg/L was only found at a 10-fold higher inoculum. Although Yeo and Livermore^36^ cite geometric mean rather than mode MICs, these parameters should approximately match given the symmetrical MIC distributions illustrated in Figure 6. As with *H. influenzae*, a plausible explanation for higher MICs is a shift towards isolates producing more β-lactamase and consequently expressing greater resistance. Again, this would require direct assays of β-lactamase-specific activity for confirmation.

The other striking features of the *M. catarrhalis* data are the differentials between the modal MICs for β-lactamase -producing and -non-producing isolates. While this ratio was as low as 2-fold for cefuroxime, confirming previous results,^36^ it rose to 16–32-fold for co-amoxiclav, cefaclor and cefotaxime, 64-fold for ceftobiprole and 512-fold for ceftaroline, compared with 2048-fold for ampicillin. The high ratio for ceftaroline is in keeping with the large inoculum effect recorded by Citron *et al*.^37^ While this phenomenon raises obvious concerns, we can find no record of ceftaroline treatment failure associated with *M. catarrhalis* although, unhelpfully, the species was not noted among the pathogens recorded in the licensing and subsequent trials.^38,39^ There also is a view that β-lactamase-producing *M. catarrhalis* can exert an ‘indirect pathogenicity’ by protecting co-present *S. pneumoniae* (or other organisms) in mixed respiratory communities,^40^ but there is no evidence that this is significant for ceftaroline which, according to the particular study and analysis, achieved non-inferiority or slight superiority to ceftriaxone in community pneumonia trials.^38^

The results of this surveillance raise few concerns about the future treatability of CA-LRTI. Although serotype-related resistance shifts are occurring in respiratory pneumococci and both BLNAR and highly amoxicillin-resistant *H. influenzae* are becoming more frequent, there is no shortage of near-universally active agents against these species. In the case of *M. catarrhalis*, there is no evidence that the β-lactamase lability of newer cephalosporins is associated with clinical failure.

## Supplementary Material

dkaf252_Supplementary_Data

## Transparency declarations

S.M., A.C. and R.A. are employees of the UKHSA and work for the Antimicrobial Resistance and Healthcare Associated Infections Reference Unit (AMRHAI), which has received financial support for conference attendance, research projects or contracted evaluations from numerous sources, including Accelerate Diagnostics, Allecra Therapeutics, AstraZeneca UK Ltd, AusDiagnostics, Basilea Pharmaceutica, Becton Dickinson Diagnostics, bioMérieux, Bio-Rad Laboratories, BSAC, Cepheid, Check-Points B.V., UK Department of Health, Enigma Diagnostics, Food Standards Agency, GlaxoSmithKline Services Ltd, Helperby Therapeutics, Henry Stewart Talks, IHMA Ltd, Innovate UK, Integra holdings, Merck Sharpe & Dohme Corp., Meiji Seika Pharma Co. Ltd, Mobidiag, Momentum Biosciences Ltd, Neem Biotech, Nordic Pharma Ltd, Norgine Pharmaceuticals, Paratek Pharmaceuticals, Roche, Smith & Nephew UK Ltd, Shionogi & Co. Ltd, Trius Therapeutics, VenatoRx Pharmaceuticals and Wockhardt Ltd. M.A. is a Trustee of the BSAC Council and is employed by MSD (UK) Limited, London, UK. M.A. has shareholdings in Merck & Co., Inc., Rahway, NJ, USA. C.L. is a trustee of the BSAC Council and is employed by Shionogi B.V. C.H. was employed by Una Health Limited and QuantuMDx Group Limited during the period of data analysis and manuscript preparation. B.J.P.: consultancy: Genpax. D.M.L.: advisory boards or *ad hoc* consultancy: AdjuTec, AstraZeneca, Centauri, Genpax, GSK, LipoVation, Paion, Shionogi, Sumitovant, Thermo Fisher and Wockhardt; paid lectures: bioMérieux, Pfizer, Shionogi and Zuellig Pharma; and relevant shareholdings or options: Genpax, GSK, Merck & Co. Inc., and Revvity amounting to <10% of portfolio value. D.M.L. also has shareholdings in Arecor, GENinCode, Probiotix, Rua Life Sciences, SkinBioTherapeutics and VericiDx (all with research/products pertinent to medicines or diagnostics) through Enterprise Investment Schemes, but has no authority to trade these holdings directly. All other authors have nothing to declare.

## Supplementary data

Figures S1 and S2 and Tables S1–S9 and Appendix are available as Supplementary data at *JAC* Online.

## References

[1] Millett ERC, Quint JK, Smeeth L et al Incidence of community-acquired lower respiratory tract infections and pneumonia among older adults in the United Kingdom: a population-based study. PLoS One 2013; 8: e75131. 10.1371/journal.pone.0075131 24040394 PMC3770598

[2] Chalmers JD, Campling J, Dicker A et al A systematic review of the burden of vaccine preventable pneumococcal disease in UK adults. BMC Pulm Med 2016; 16: 77. 10.1186/s12890-016-0242-0 27169895 PMC4864929

[3] Danino D, Ben-Shimol S, van der Beek BA et al Decline in pneumococcal disease in young children during the coronavirus disease 2019 (COVID-19) pandemic in Israel associated with suppression of seasonal respiratory viruses, despite persistent pneumococcal carriage: a prospective cohort study. Clin Infect Dis 2022; 75: e1154–64. 10.1093/cid/ciab1014 34904635 PMC8754767

[4] Brueggemann AB, Jansen van Rensburg MJ, Shaw D et al Changes in the incidence of invasive disease due to *Streptococcus pneumoniae*, *Haemophilus influenzae*, and *Neisseria meningitidis* during the COVID-19 pandemic in 26 countries and territories in the Invasive Respiratory Infection Surveillance Initiative: a prospective analysis of surveillance data. Lancet Digit Health 2021; 3: e360–70. 10.1016/S2589-7500(21)00077-7 34045002 PMC8166576

[5] Gadsby NJ, Musher DM. The microbial etiology of community-acquired pneumonia in adults: from classical bacteriology to host transcriptional signatures. Clin Microbiol Rev 2022; 35: e0001522. 10.1128/cmr.00015-22 36165783 PMC9769922

[6] Blasi F, Garau J, Medina J et al Current management of patients hospitalized with community-acquired pneumonia across Europe: outcomes from REACH. Respir Res 2013; 14: 44. 10.1186/1465-9921-14-44 23586347 PMC3644236

[7] Rello J, Allam C, Ruiz-Spinelli A et al Severe legionnaires’ disease. Ann Intensive Care 2024; 14: 51. 10.1186/s13613-024-01252-y 38565811 PMC10987467

[8] Ladhani SN, Slack MPE, Andrews NJ et al Invasive pneumococcal disease after routine pneumococcal conjugate vaccination in children, England and Wales. Emerg Infect Dis 2013; 19: 61–8. 10.3201/eid1901.120741 23259937 PMC3557991

[9] Miller E, Andrews NJ, Waight PA et al Herd immunity and serotype replacement 4 years after seven-valent pneumococcal conjugate vaccination in England and Wales: an observational cohort study. Lancet Infect Dis 2011; 11: 760–8. 10.1016/S1473-3099(11)70090-1 21621466

[10] Tsaban G, Ben-Shimol S. Indirect (herd) protection, following pneumococcal conjugated vaccines introduction: a systematic review of the literature. Vaccine 2017; 35: 2882–91. 10.1016/j.vaccine.2017.04.032 28449971

[11] Horner C, Reynolds R, Mushtaq S et al Trends of serotypes and resistance among *Streptococcus pneumoniae* in the UK and Ireland (1999–2019). J Antimicrob Chemother 2025; 80 (Suppl 4): iv72–iv86. 10.1093/jac/dkaf253 PMC12556646 41140271

[12] Allen M, Reynolds R, Mushtaq S, et al The British Society for Antimicrobial Chemotherapy Resistance Surveillance Project: methods and limitations. J Antimicrob Chemother 2025; 80 (Suppl 4): iv7–iv21. 10.1093/jac/dkn348 18819978

[13] Bischoff D . BLINDSCHEMES: Stata module to provide graph schemes sensitive to color vision deficiency. Statistical Software Components S458251, Boston College Department of Economics, revised 07 Aug 2020. https://ideas.repec.org/cgi-bin/refs.cgi.

[14] Hirai J, Kinjo T, Koga T et al Clinical characteristics of community-acquired pneumonia due to *Moraxella catarrhalis* in adults: a retrospective single-centre study. BMC Infect Dis 2020; 20: 821. 10.1186/s12879-020-05564-9 33172398 PMC7653842

[15] Gilchrist M, Barr D, Drummond F et al Outpatient parenteral antimicrobial therapy (OPAT) in the UK: findings from the BSAC National Outcomes Registry (2015-19). J Antimicrob Chemother 2022; 77: 1481–90. 10.1093/jac/dkac047 35187565

[16] Horner C, Mushtaq S, Livermore DM et al Activity of ceftaroline versus ceftobiprole against staphylococci and pneumococci in the UK and Ireland: analysis of BSAC surveillance data. J Antimicrob Chemother 2020; 75: 3239–43. 10.1093/jac/dkaa306 32728710

[17] EUCAST . Clinical breakpoints and dosing of antibiotics. https://www.eucast.org/clinical_breakpoints/.

[18] Lansbury L, Lawrence H, McKeever TM et al Pneumococcal serotypes and risk factors in adult community acquired pneumonia 2018–20: a multicentre UK cohort study. Lancet Reg Health Eur 2024; 37: 100812. https://www.thelancet.com/journals/lanepe/article/PIIS2666-7762(23)00231-4/fulltext. 10.1016/j.lanepe.2023.100812 38170136 PMC10758948

[19] Horner C, Mushtaq S, Allen M et al Are resistance rates among bloodstream isolates a good proxy for other infections? Analysis from the BSAC Resistance Surveillance Programme. J Antimicrob Chemother 2021; 76: 1822–31. 10.1093/jac/dkab096 33822968

[20] Roberts MC . Resistance to macrolide, lincosamide, streptogramin, ketolide, and oxazolidinone antibiotics. Mol Biotechnol 2004; 28: 47–62. 10.1385/MB:28:1:47 15456963

[21] Sheppard C, Fry NK, Mushtaq S et al Rise of multidrug-resistant non-vaccine serotype 15A *Streptococcus pneumoniae* in the United Kingdom, 2001 to 2014. Euro Surveill 2016; 21: 30423. 10.2807/1560-7917.ES.2016.21.50.30423 28006650 PMC5291132

[22] Bozdogan B, Bogdanovich T, Kosowska K et al Macrolide resistance in *Streptococcus pneumoniae*: clonality and mechanisms of resistance in 24 countries. Curr Drug Targets Infect Disord 2004; 4: 169–76. 10.2174/1568005043340821 15379728

[23] Birtles A, Virgincar N, Sheppard CL et al Antimicrobial resistance of invasive *Streptococcus pneumoniae* isolates in a British district general hospital: the international connection. J Med Microbiol 2004; 53: 1241–6. 10.1099/jmm.0.45763-0 15585504

[24] Anon . European Antimicrobial Resistance Surveillance Network (EARS-Net). European Centre for Disease Prevention and Control. https://www.ecdc.europa.eu/en/about-us/partnerships-and-networks/disease-and-laboratory-networks/ears-net.

[25] Matic V, Bozdogan B, Jacobs MR et al Contribution of beta-lactamase and PBP amino acid substitutions to amoxicillin/clavulanate resistance in beta-lactamase-positive, amoxicillin/clavulanate-resistant *Haemophilus influenzae*. J Antimicrob Chemother 2003; 52: 1018–21. 10.1093/jac/dkg474 14585854

[26] Heinz E . The return of Pfeiffer’s bacillus: rising incidence of ampicillin resistance in *Haemophilus influenzae*. Microb Genom 2018; 4: e000214. 10.1099/mgen.0.000214 30207515 PMC6202453

[27] Kim I-S, Ki C-S, Kim S et al Diversity of ampicillin resistance genes and antimicrobial susceptibility patterns in *Haemophilus influenzae* strains isolated in Korea. Antimicrob Agents Chemother 2007; 51: 453–60. 10.1128/AAC.00960-06 17116681 PMC1797734

[28] Ito M, Hotomi M, Maruyama Y et al Clonal spread of beta-lactamase-producing amoxicillin-clavulanate-resistant (BLPACR) strains of non-typeable *Haemophilus influenzae* among young children attending a day care in Japan. Int J Pediatr Otorhinolaryngol 2010; 74: 901–6. 10.1016/j.ijporl.2010.05.008 20846501

[29] Skaare D, Anthonisen IL, Kahlmeter G et al Emergence of clonally related multidrug resistant *Haemophilus influenzae* with penicillin-binding protein 3-mediated resistance to extended-spectrum cephalosporins, Norway, 2006 to 2013. Euro Surveill 2014; 19: 20986. 10.2807/1560-7917.ES2014.19.49.20986 25523969

[30] Månsson V, Skaare D, Riesbeck K et al The spread and clinical impact of ST14CC-PBP3 Type IIb/A, a clonal group of non-typeable *Haemophilus influenzae* with chromosomally mediated β-lactam resistance—a prospective observational study. Clin Microbiol Infect 2017; 23: 209.e1–209.e7. 10.1016/j.cmi.2016.11.006 27852000

[31] García-Cobos S, Campos J, Lázaro E et al Ampicillin-resistant non-beta-lactamase-producing *Haemophilus influenzae* in Spain: recent emergence of clonal isolates with increased resistance to cefotaxime and cefixime. Antimicrob Agents Chemother 2007; 51: 2564–73. 10.1128/AAC.00354-07 17470649 PMC1913223

[32] Honda H, Sato T, Shinagawa M et al Multiclonal expansion and high prevalence of β-lactamase-negative *Haemophilus influenzae* with high-level ampicillin resistance in Japan and susceptibility to quinolones. Antimicrob Agents Chemother 2018; 62: e00851-18. 10.1128/AAC.00851-18 29987153 PMC6125502

[33] Jakubu V, Malisova L, Musilek M et al Characterization of *Haemophilus influenzae* strains with non-enzymatic resistance to β-lactam antibiotics caused by mutations in the PBP3 gene in the Czech Republic in 2010–2018. Life (Basel) 2021; 11: 1260. 10.3390/life11111260 34833138 PMC8624647

[34] Takahata S, Ida T, Senju N et al Horizontal gene transfer of *ftsI*, encoding penicillin-binding protein 3, in *Haemophilus influenzae*. Antimicrob Agents Chemother 2007; 51: 1589–95. 10.1128/AAC.01545-06 17325223 PMC1855551

[35] Fung CP, Powell M, Seymour A et al The antimicrobial susceptibility of *Moraxella catarrhalis* isolated in England and Scotland in 1991. J Antimicrob Chemother 1992; 30: 47–55. 10.1093/jac/30.1.47 1429336

[36] Yeo SF, Livermore DM. Effect of inoculum size on the in-vitro susceptibility to beta-lactam antibiotics of *Moraxella catarrhalis* isolates of different beta-lactamase types. J Med Microbiol 1994; 40: 252–5. 10.1099/00222615-40-4-252 8151675

[37] Citron DM, Warren YA, Tyrrell KL et al Activity of ceftaroline against aerobic Gram-positive and Gram-negative pathogens: effect of test method variability. ISRN Microbiol 2011; 2011: 787290. 10.5402/2011/787290 23724310 PMC3658583

[38] Carreno JJ, Lodise TP. Ceftaroline fosamil for the treatment of community-acquired pneumonia: from FOCUS to CAPTURE. Infect Dis Ther 2014; 3: 123–32. 10.1007/s40121-014-0036-8 25193094 PMC4269637

[39] Lan S-H, Chang S-P, Lai C-C et al Efficacy and safety of ceftaroline for the treatment of community-acquired pneumonia: a systemic review and meta-analysis of randomized controlled trials. J Clin Med 2019; 8: 824. 10.3390/jcm8060824 31181859 PMC6617040

[40] Budhani RK, Struthers JK. Interaction of *Streptococcus pneumoniae* and *Moraxella catarrhalis*: investigation of the indirect pathogenic role of beta-lactamase-producing moraxellae by use of a continuous-culture biofilm system. Antimicrob Agents Chemother 1998; 42: 2521–6. 10.1128/AAC.42.10.2521 9756750 PMC105877

