# Supplementary material for: Antimicrobial resistance among agents of community-associated lower respiratory tract infection in the UK and Ireland: trends from 1999/2000 to 2018/2019
Source: J Antimicrob Chemother. 2025 Oct 27;80(Suppl 4):iv60–71. doi: 10.1093/jac/dkaf252 (PMC12555909; doi:10.1093/jac/dkaf252)
Supplement: dkaf252_Supplementary_Data [file dkaf252_supplementary_data.pdf]

Antimicrobial resistance among agents of community-associated lower respiratory tract infection in the UK and Ireland: trends from 1999/2000 to 2018/2019

SUPPLEMENTARY INFORMATION

Methods for the BSAC Resistance Surveillance Project are described in detail in a companion paper.[1] Breakpoints and ECOFFs (Epidemiological Cut-offs) listed in Tables S3–S5 are from EUCAST tables of breakpoints (v12.0) and related guidance at the time of analysis (https://www.eucast.org), specifically:

European Committee on Antimicrobial Susceptibility Testing. **Breakpoint tables** for interpretation of MICs and zone diameters. Version 12.0, valid from 2022-01-01.". https://www.eucast.org; https://www.eucast.org/fileadmin/src/media/PDFs/EUCAST_files/Breakpoint_tables/v_12.0_Breakpoint_Tables.pdf. Accessed 20 January 2025

European Committee on Antimicrobial Susceptibility Testing. Guidance document: **EUCAST breakpoints in brackets** 1 December, 2021. Available at: https://www.eucast.org/fileadmin/src/media/PDFs/EUCAST_files/Guidance_documents/Breakpoints_in_brackets.pdf /. Accessed 13 January 2025.

European Committee on Antimicrobial Susceptibility Testing. Data from the EUCAST MIC distribution website. https://www.eucast.org/mic_and_zone_distributions_and_ecoffs; https://mic.eucast.org/; https://mic.eucast.org/search/.

Contents

BSAC Community-associated LRTI resistance surveillance – isolates and tests 3

Table S1. Isolate collection quotas and targets – BSAC CA-LRTI surveillance 3

Table S2. Actual numbers of CA-LRTI isolates tested, and centres contributing by year – BSAC respiratory surveillance 3

Data exclusions, interpretation, compliance and change of central testing laboratory 4

Table S3. *S. pneumoniae*: antibiotics analysed – years included, N of isolates, resistance breakpoints and mode MICs 4

Table S4. *H. influenzae*: antibiotics analysed – years included, N of isolates, resistance breakpoints and mode MICs 5

Table S5. *M. catarrhalis*: antibiotics analysed – years included, N of isolates, resistance breakpoints and mode MICs 6

BSAC community-associated LRTI resistance surveillance – patient characteristics 7

Table S6. CA-LRTI percentage of male patients by organism group 7

Table S7. Patient age: summary measures by organism group 7

Figure S1. Patient age: histograms and trends by organism group 8

Figure S2. Trends in care setting by organism group 9

Table S8. Proportion of ICU patients by organism group, among those in hospital (≤48 hours) 10

Table S9. Specimen types: % by organism 10

References **11**
APPENDIX – MIC distributions **12**
S. pneumoniae 12
H. influenzae 13
M. catarrhalis 14

BSAC Community-associated LRTI resistance surveillance – isolates and tests

Table S1. Isolate collection quotas and targets – BSAC CA-LRTI surveillance

Annual collection periods[1]	Target N of centres	*S. pneumoniae* / *H. influenzae* Quota per lab per species	Target: total per species	*M. catarrhalis* Quota/lab	Target
1999/00–2007/08	20	50	1000	25	500
2008/09–2009/10	20	25	500	13	260
2010/11–2014/15	40	14	560	7	280
2015/16–2018/19	25	20	500	10	250

[1] 1 October – 30 April from 1999/2000 to 2007/08; 1 October – 30 September from 2008/09 to 2018/19.

Collections per participating centre per season averaged 72% of quota for *S. pneumoniae*, 89% for *H. influenzae* and 84% for *M. catarrhalis*.

Table S2. Actual numbers of CA-LRTI isolates tested, and centres contributing by year – BSAC respiratory surveillance

Collection season	Collection period	N of Centres[1]	N of isolates *S. pneumoniae*	*H. influenzae*	*M. catarrhalis*[2]
1999/2000	October–April	20	661	936	421
2000/01	October–April	20	667	958	424
2001/02	October–April	22	699	916	418[(2)]
2002/03	October–April	22	772	926	438
2003/04	October–April	22	785	899	422[(2)]
2004/05	October–April	21	750	888	403
2005/06	October–April	22	749	942	415
2006/07	October–April	21	727	906	428
2007/08	October–April	23	809	1,004	461
2008/09	October–September	22	451	501	253
2009/10	October–September	23	480	528	270
2010/11	October–September	39	420	516	236
2011/12	October–September	38	383	489	236
2012/13	October–September	34	345	416	223
2013/14	October–September	39	375	482	207
2014/15	October–September	39	429	508	239
2015/16	October–September	24	358	448	203
2016/17	October–September	25	345	429	205
2017/18	October–September	24	325	416	190
2018/19	October–September	24	351	418	217
Total		(79)	10,881	13,526	6,309

[1] Number of sites that actually contributed any CA-LRTI isolates in that season; not all necessarily contributed isolates of all organism groups.
[2] In 2001/02 and 2003/04, *M. catarrhalis* was tested only for microbial identity, β-lactamase production (with nitrocefin) and fluoroquinolone susceptibility (predicted with nalidixic acid).

Data exclusions, interpretation, compliance and change of central testing laboratory

The recommended inoculum for MIC determinations of β-lactams against *M. catarrhalis* changed to 10^6 CFU/spot early in the surveillance period.[2,3] Accordingly, results for amoxicillin, cefaclor, cefuroxime and cefotaxime in 1999/2000, obtained with inocula of 10^4 CFU/spot, are excluded. All results shown are for 10^6 CFU/spot, which was used from 1999/2000 for ampicillin and co-amoxiclav, and from 2000/01 for all β-lactams.

Co-amoxiclav was tested using a 2:1 ratio of amoxicillin:clavulanate from 1999/2000 to 2012/13.[1] This method was then abandoned and its results are excluded from graphs of MIC distributions, as incompatible with the later recommendation to test with a fixed 2 mg/L clavulanate concentration.[4] However, these results still allow good estimates of percent susceptibility/resistance in *H. influenzae* and *M. catarrhalis* at the breakpoints used and are retained e.g. in Tables S4 and S5.

A few isolates were included despite departures from protocol requirements for care setting and sample type. These are noted where relevant (see Figure S2, Table S9).

Central testing was at GR Micro, London (later Quotient Bioresearch, then LGC, Fordham, UK) in 1999/2000–2012/13 and at the Antimicrobial Resistance and Healthcare-Associated Infections Reference Unit (AMRHAI) of Public Health England (later the UK Health Security Agency), Colindale, London, in 2013/14–2018/19.

Table S3. *S. pneumoniae*: antibiotics analysed – years included, N of isolates, resistance breakpoints and mode MICs

Antimicrobial	Seasons included	N of seasons	Break-point R > mg/L	*S. pneumoniae* N of isolates	*S. pneumoniae* Mode MIC mg/L
Amoxicillin[1]	1999/2000–2018/19	20	1	10881	0.015‡
Cefaclor	1999/2000–2004/05	6	0.5	4334	0.25‡
Cefotaxime	1999/2000–2018/19	20	2	10881	0.015
Ceftaroline	2016/17-2018/19	3	0.25	1021	0.008
Ceftobiprole	2011/12–2018/19	8	0.5	2911	0.015‡
Cefuroxime	1999/2000–2013/14	15	1	9073	0.015‡
Clarithromycin	1999/2000–2002/03	4	0.5	2799	0.06‡
Clindamycin[2]	1999/2000–2018/19	20	0.5	10881	0.12‡
Erythromycin	1999/2000–2018/19	20	0.5	10881	0.12‡
Levofloxacin	1999/2000–2000/01; 2018/19	3	2	1679	1
Moxifloxacin	1999/2000–2003/04; 2015/16	6	0.5	4201	0.12‡
Penicillin[3]	1999/2000–2018/19	20	2	10881	0.015‡
Tetracycline	1999/2000–2018/19	20	2	10881	0.25‡

‡ Most frequent MIC, but distribution was clearly bi- or multi-modal: refer to plot in Appendix.

[1] Amoxicillin has no intravenous breakpoint, but this oral breakpoint matches the i.v. breakpoint for ampicillin.

[2] Clindamycin resistance is reported at face value using this (EUCAST) breakpoint. Inducible resistance was not tested for *S. pneumoniae*.

[3] Isolates with penicillin MIC >0.06 mg/L but ≤2 mg/L are classified I (“susceptible, increased exposure”).

Table S4. *H. influenzae*: antibiotics analysed – years included, N of isolates, resistance breakpoints and mode MICs

Antimicrobial	Seasons included	N of seasons	Break-point R > mg/L	*H. influenzae*	
				N of isolates	Mode MIC mg/L
Amoxicillin	1999/2000–2018/19	20	2	13526	0.5‡
Co-amoxiclav[1]	2013/14–2018/19	6	2	2701	0.5
Ampicillin	1999/2000–2014/15	16	1	11815	0.25‡
Cefaclor	1999/2000-2004/05	6	-	5523	2
Cefotaxime	1999/2000–2018/19	20	0.12	13526	0.015
Ceftaroline	2016/17–2018/19	3	0.03	1263	0.008
Ceftobiprole	2011/12–2018/19	8	-	3606	0.06
Cefuroxime	1999/2000–2013/14	15	2	11307	0.5
Ciprofloxacin	1999/2000–2018/19	20	0.06	13526	0.008‡
Clarithromycin[2]	1999/2000–2002/03	4	32	3736	4
Ertapenem	2004/05–2006/07	3	0.5	2736	0.03
Erythromycin[2]	1999/2000–2014/15	16	16	11815	4‡
Levofloxacin	1999/2000–2000/01; 2018/19	3	0.06	2312	0.015‡
Minocycline	2004/05–2012/13	9	1	6190	0.25
Moxifloxacin	1999/2000–2003/04; 2015/16	6	0.12	5083	0.03
Tetracycline	1999/2000–2018/19	20	2	13526	0.5‡
Tigecycline	2004/05–2012/13	9	-	6190	0.25
Trimethoprim	1999/2000–2014/15	16	-	11815	0.12‡

‡ Most frequent MIC, but distribution was clearly bi- or multi-modal: refer to plot in Appendix.

[1] As tested with fixed 2 mg/L clavulanate. From 1999/2000 to 2012/13, the mode MIC for co-amoxiclav, as tested against 10825 isolates using the now abandoned 2:1 amoxicillin:clavulanate ratio formulation also was 0.5 mg/L, which is predictable, given that the great majority of *H. influenzae* lack β-lactamase. The change in testing modality effectively raised the breakpoint from >2+1 to >2+2 mg/L so the resistance rate recorded by ratio testing, 1.1%, is an upper limit for what would have been found in that period if tested with the later fixed 2 mg/L clavulanate concentration.

[2] Clinical evidence for the efficacy of macrolides in *H. influenzae* respiratory infections is conflicting due to high spontaneous cure rates.[4] The 'breakpoints' shown are ECOFFs, used to detect strains with acquired resistance.

Table S5. *M. catarrhalis*: antibiotics analysed – years included, N of isolates, resistance breakpoints and mode MICs

Antimicrobial	Seasons included	N of seasons	Break-point R> mg/L	*M. catarrhalis*	
				N of isolates	Mode MIC mg/L
Amoxicillin	2000/01; 2002/03; 2004/05	3	-	1265	16‡
Co-amoxiclav[2]	2013/14–2018/19	6	1	1261	0.12‡
Ampicillin	1999/00–2000/01; 2002/03; 2004/05	4	-	1686	8–16‡
Cefaclor	2000/01; 2002/03; 2004/05;	3	-	1265	4
Cefotaxime	2000/01; 2002/03; 2004/05; 2014/15–2018/19	8	2	2319	0.5‡
Ceftaroline	2016/17–2018/19	3	-	612	4
Ceftobiprole	2011/12–2018/19	8	-	1720	1
Cefuroxime	2000/01; 2002/03; 2004/05; 2005/06–2013/14	12	8	3994	1
Ciprofloxacin	1999/00–2000/01; 2002/03; 2004/05–2018/19	18	0.12	5469	0.03‡
Clarithromycin	1999/00–2000/01; 2002/03;	3	0.5	1283	0.03
Erythromycin	1999/00–2000/01; 2002/03; 2004/05–2018/19	18	0.5	5469	0.06
Levofloxacin	1999/00–2000/01; 2018/19	3	0.12	1062	0.06
Minocycline	2004/05–2012/13	9	1	2925	0.12
Moxifloxacin	1999/00–2000/01; 2002/03; 2015/16	4	0.25	1486	0.06
Tetracycline	1999/00–2000/01; 2002/03; 2004/05–2018/19	18	2	5469	0.5
Tigecycline	2004/05–2012/13	9	-	2925	0.12
Trimethoprim	1999/00–2000/01; 2002/03; 2004/05	4	-	1686	16

‡ Most frequent MIC, but distribution was clearly bi- or multi-modal: refer to plot in Appendix.
[1] As tested with a fixed 2 mg/L clavulanate. From 1999/2000–2012/13, the mode MIC for co-amoxiclav tested against 4208 isolates using the now abandoned 2:1 amoxicillin:clavulanate ratio formulation was 0.25 mg/L and estimated resistance was ≤0.1%: there was a single resistant isolate with an MIC of 2+1 mg/L amoxicillin + clavulanate; it might have been inhibited (and therefore judged susceptible) at 1+ 2 mg/L in fixed concentration testing.

Note: *M. catarrhalis* was tested only for microbial identity, β-lactamase (with nitrocefin) and fluoroquinolone susceptibility (as predicted with nalidixic acid) in 2001/02 and 2003/04.

BSAC community-associated LRTI resistance surveillance – patient characteristics

Sex

The proportion of male patients was fairly stable at close to 50% for *H. influenzae* and *M. catarrhalis* but, for *S. pneumoniae*, it fell gradually from around 60% in the first few seasons to near 50% in the last few.

Table S6. CA-LRTI percentage of male patients by organism group

Organism group	N[1]	Male, %
S. pneumoniae	10878	55.1
H. influenzae	13519	51.9
M. catarrhalis	6307	48.8

[1] Number of isolates with sex data. (Missing: 12/30716, <0.1%)

Age

The modal age groups were 60–69 and 70–79 years. *M. catarrhalis* was more strongly associated with older age groups than were *S. pneumoniae* or *H. influenzae*. *H. influenzae* showed an increasing proportion of patients aged ≥65 over the surveillance period. Infants under 1 year of age together with 1-year-olds formed a distinct but small group for all three organisms.

Table S7. Patient age: summary measures by organism group

		Quartiles, years			Isolates in age group shown, %		
Organism group	N[1]	Q1	Q2	Q3	<1 year	≥65 years	≥80 years
S. pneumoniae	10872	47	63	73	1.8	46.4	11.6
H. influenzae	13511	49	64	73	1.0	48.0	10.6
M. catarrhalis	6301	54	67	76	2.0	55.4	16.0

[1] Number of isolates with age data. (Missing: 32/30716 = 0.1%)

Patients aged ≥80 years are an older subset of those who are ≥65 years old.

Figure S1. Patient age: histograms and trends by organism group

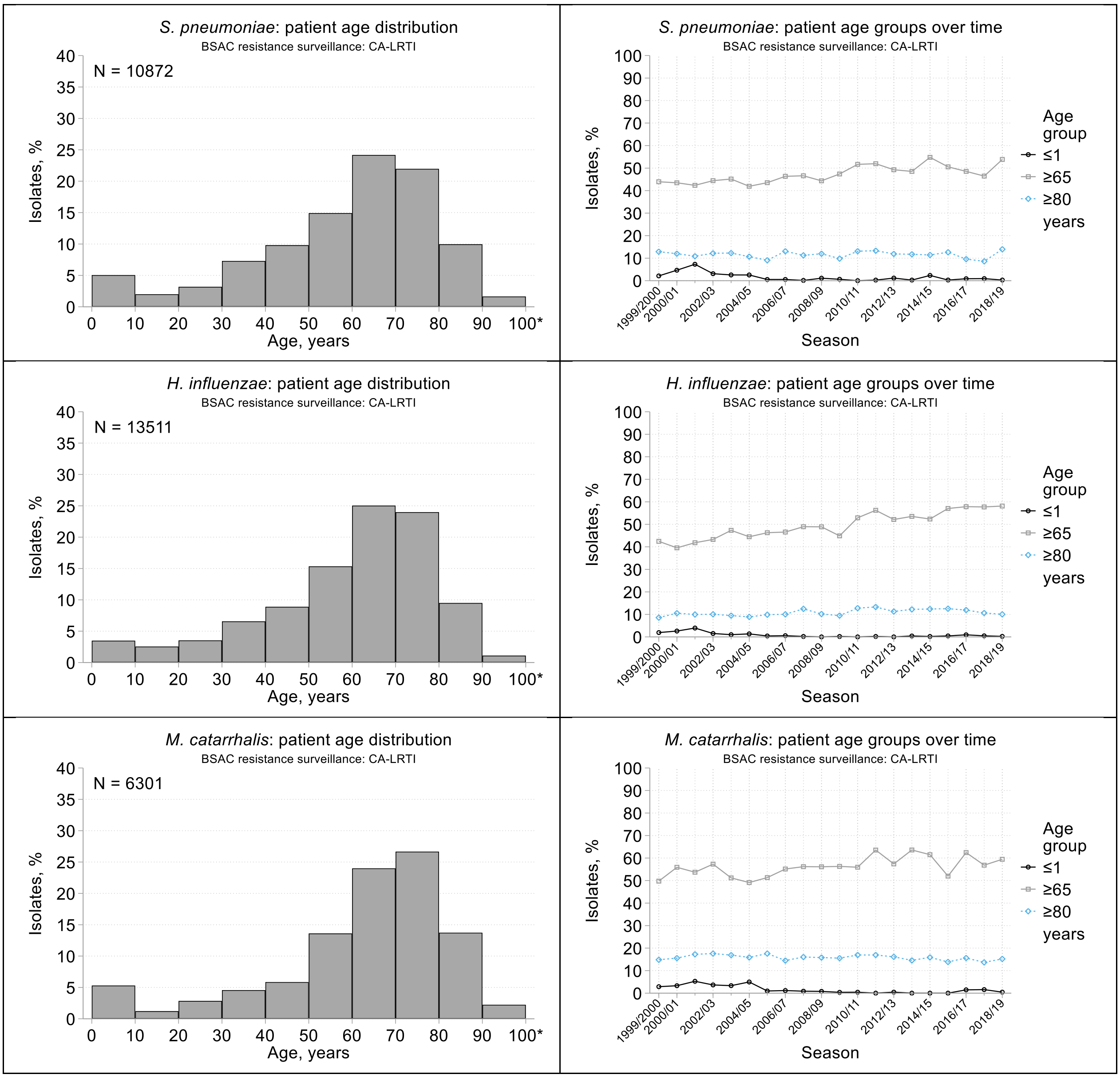

* The final bar includes all patients aged ≥90 years.

.

Care setting – hospitalised (≤48 hours) at time of sample vs community/outpatient settings

Samples from patients hospitalised for >48 hours were excluded by design, with >99.9% compliance; 19 isolates (<0.1%) were included in analyses despite breaching this condition, 14 of them from a single centre in 2018/19. Care setting data were obtained for 99.8% of the 30,716 isolates collected. A single laboratory's 2002/03 collection (18 *S. pneumoniae*, 19 *H. influenzae*, 10 *M. catarrhalis*) accounted for 47 of the 62 lacking data; they gave an assurance that none were from patients in hospital >48 hours.

Outpatients were grouped with hospital inpatients (≤48 hours) until 2012/13; afterwards, from 2013/14, they were grouped with non-hospital settings (general practice, nursing homes and similar). This step change in recording cannot explain the progressive fall in the proportion of isolates from inpatients – from around 60% to around 10% – between 2003/04 and 2014/15 for all three species (Figure S2).

Figure S2. Trends in care setting by organism group

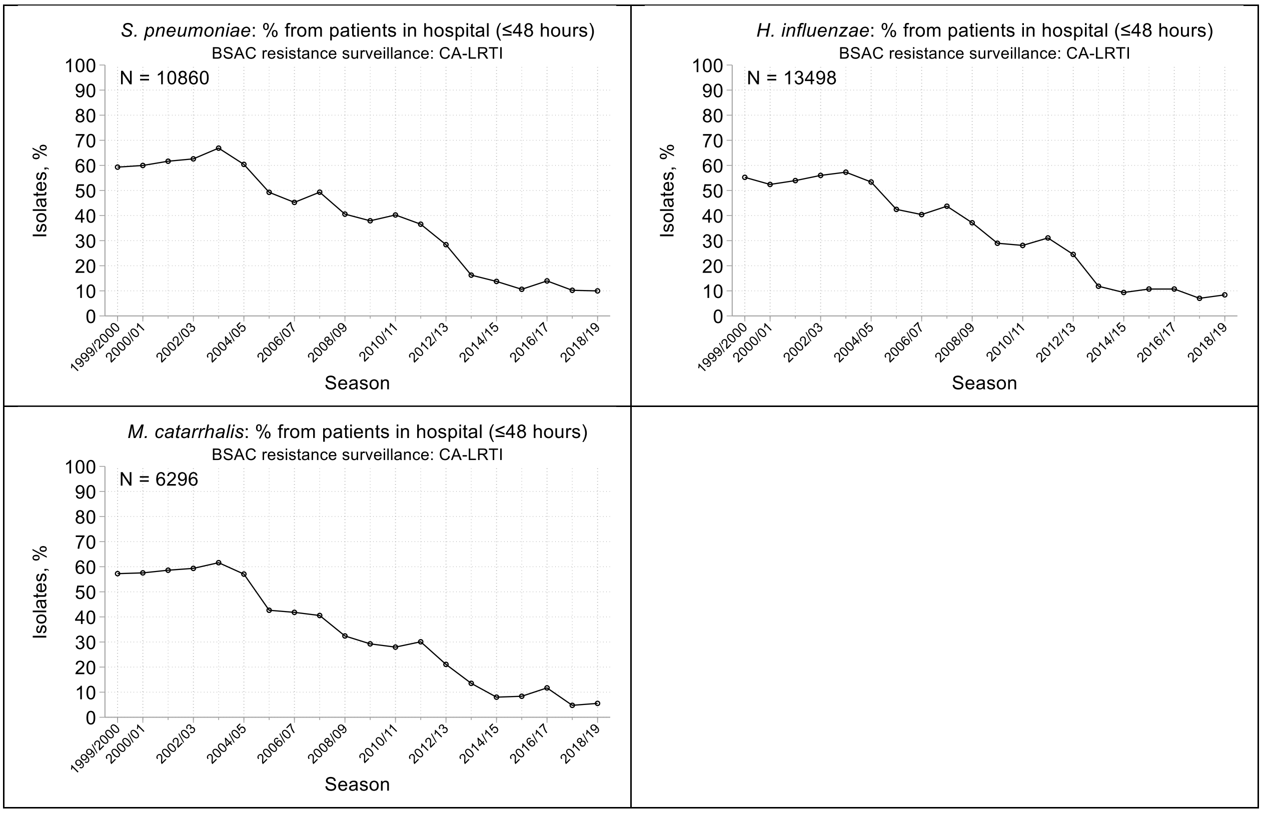

Care setting: intensive/critical care speciality (ICU)

Hospital speciality data were collected only from 2013/14, by which time only around 10% of samples were from hospital inpatients (Figure S2). Among this relatively small number, *S. pneumoniae* had the highest proportion from ICU patients (21%).

Table S8. Proportion of ICU patients by organism group, among those in hospital (≤48 hours)

Organism group	N[1]	ICU, %
S. pneumoniae	274	21.1
H. influenzae	262	14.4
M. catarrhalis	109	12.4

[1] Number of CA-LRTI isolates from hospital inpatients with speciality/ICU data, 2013/14–2018/19. (Missing: 94/645 = 14.6%.)
Caveat: The tabulated 'overall' figures do not represent any particular year and the ICU proportion changed over time.

Specimen type

By far the most frequent specimen type was sputum, accounting for 92–95% of isolates of each of the three species, with no apparent trend over time. The next-most-frequent specimen type was bronchoalveolar lavage, ranging from 1.9% for *M. catarrhalis* to 3.8% for *S. pneumoniae*.

Table S9. Specimen types: % by organism

		Specimen type, %			
Organism group	N[1]	Sputum	Tracheal[2]	BAL[3]	Other[4,5]
S. pneumoniae	10851	92.4	1.1	3.8	2.7
H. influenzae	13468	95.0	0.7	2.8	1.6
M. catarrhalis	6285	94.9	0.8	1.9	2.4

[1] Number of isolates with data for specimen type. One collecting centre in 1999/2000 did not record the specimen type for its collection of 18 *S. pneumoniae*, 50 *H. influenzae* and 23 *M. catarrhalis* isolates but gave assurance that all 91 were from lower respiratory samples. They are excluded from this table but included in other analyses. Apart from this special case, specimen data were missing for only 12 *S. pneumoniae*, 8 *H. influenzae* and 1 *M. catarrhalis* (overall, <0.1%).
[2] Tracheal/endotracheal secretions/aspirates/tips.
[3] Bronchoalveolar lavage.
[4] The 653 isolates from 'Other' specimen types included 439 from upper respiratory tract samples such as nasopharyngeal secretions/aspirates, of which most (407) were submitted and accepted in 1999/2000–2004/05, when they accounted for 169/4316 (3.9%) of *S. pneumoniae*, 122/5473 (2.2%) of *H. influenzae* and 116/2503 (4.6%) of *M. catarrhalis*. These types were actively discouraged in later seasons as not distinctly LRTI, and contributed only 32/18312 (<0.2%) of collected isolates over the subsequent seasons.
[5] The remaining 214 isolates (0.7% of all those with data) were recorded with various 'Other' LRTI specimen types, including bronchial washings/aspirates and chest drains.

References

1. Allen M, Reynolds R, Mushtaq S *et al*. The British Society for Antimicrobial Chemotherapy Resistance Surveillance Project: methods and limitations. *J Antimicrob Chemother* 2025; **80** (Suppl 4): iv7–iv21.

2. Andrews JM. Determination of minimum inhibitory concentrations. *J Antimicrob Chemother* 2002; **49**: 1049.

3. Andrews JM. Determination of minimum inhibitory concentrations. *J Antimicrob Chemother* 2001; **48 Suppl 1**: 5–16.

4. Anon. eucast: Clinical breakpoints and dosing of antibiotics. Available at: https://www.eucast.org/clinical_breakpoints/. Accessed November 2, 2022.

APPENDIX – MIC distributions

These graphs are presented as thumbnails for reasons of space. Please zoom in to read.
The red vertical lines show EUCAST v12.0 (2022) breakpoint(s)[4] or, if used for analysis in the absence of breakpoints, ECOFFs – see Tables S4–S11. Where two lines are shown, they indicate the susceptible (S ≤) and resistant (R >) breakpoints; MICs between these bounds are designated I "susceptible, increased exposure". More commonly, there is a single line because the S and R breakpoints are coincident and there is no I category. Occasionally, noted below, there is no S category, and the single line demarcates R from I.

Some distributions were affected by excessive censoring due to testing of restricted concentration ranges, usually in earlier years. These years' data are omitted, as noted in the affected plots, to show the true range more accurately.

Collection years and number of isolates are noted within each plot. The MIC axes all span ≤0.001 to ≥1024 mg/L, with labelled values showing the range of MICs actually observed in those years.

Combinations lacking S category
S. pneumoniae: cefaclor, levofloxacin

S. pneumoniae

S. pneumoniae

Amoxicillin

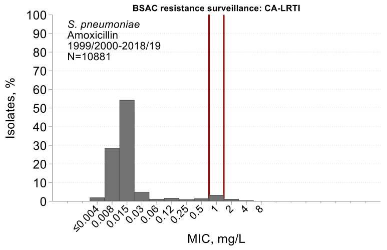

Cefaclor

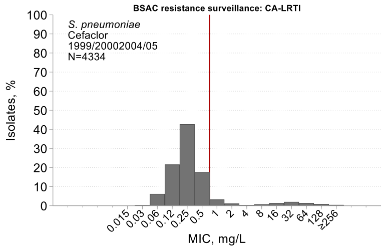

Cefotaxime

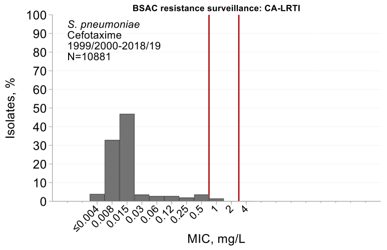

Ceftaroline

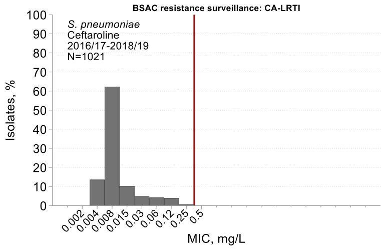

Ceftobiprole

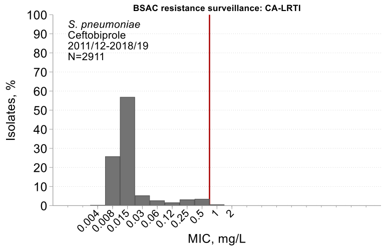

Cefuroxime

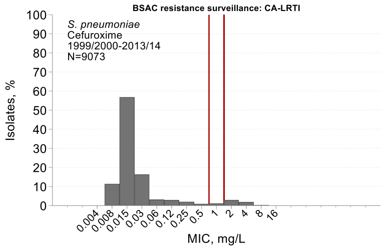

Clarithromycin

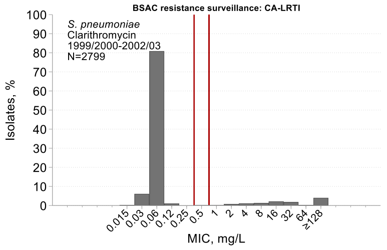

Clindamycin

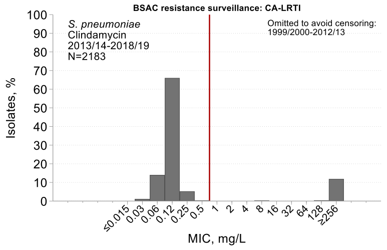

Erythromycin

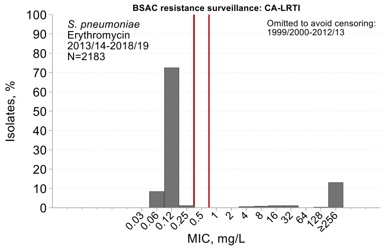

S. pneumoniae		
**Levofloxacin** 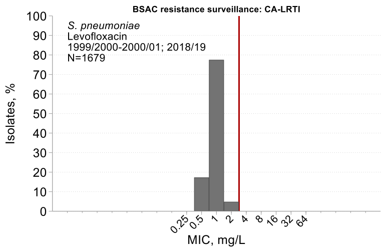	**Moxifloxacin** 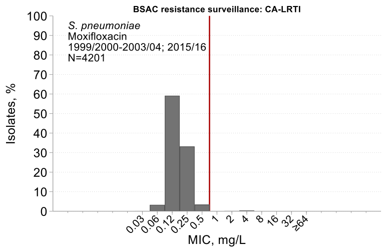	**Penicillin** 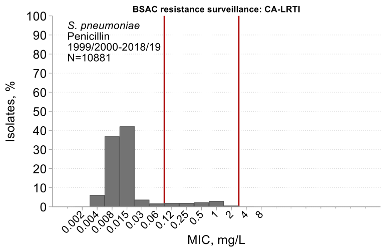
**Tetracycline** 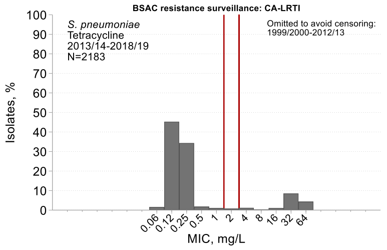		

H. influenzae

H. influenzae		
**Amoxicillin** 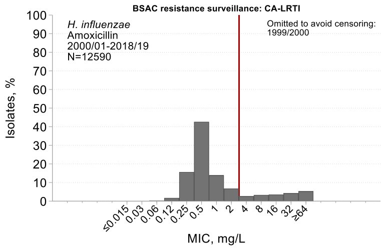	**Ampicillin** 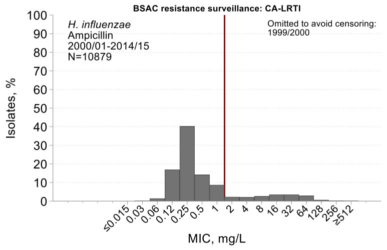	**Co-amoxiclav (2:1 ratio)** 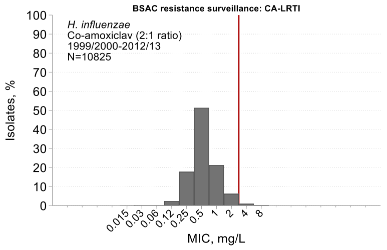
**Co-amoxiclav (2 mg/L clavulanate)** 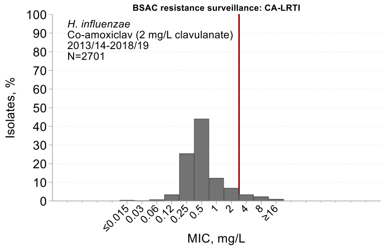	**Cefaclor** 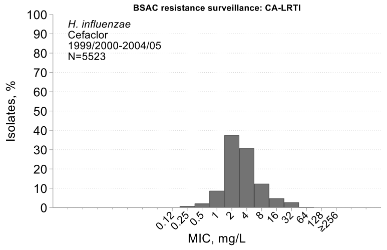	**Cefotaxime** 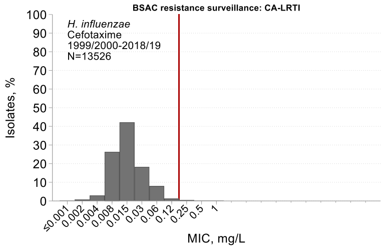
**Ceftaroline** 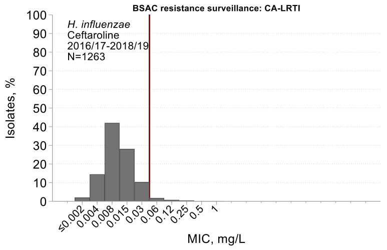	**Ceftobiprole** 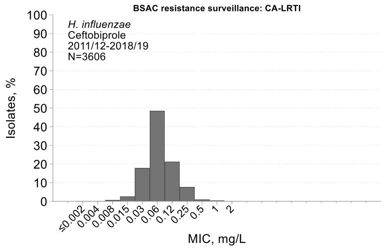	**Cefuroxime** 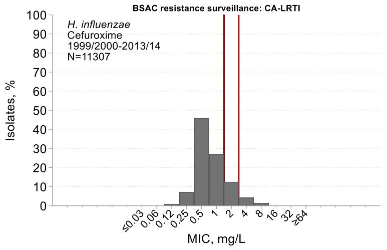

H. influenzae

Ciprofloxacin

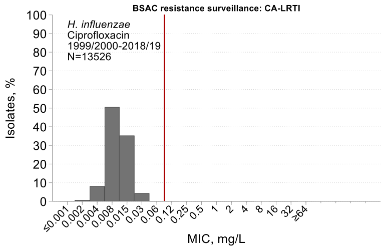

Clarithromycin

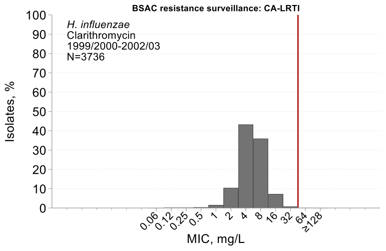

Ertapenem

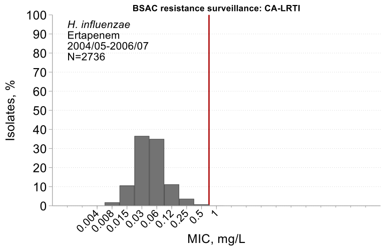

Erythromycin

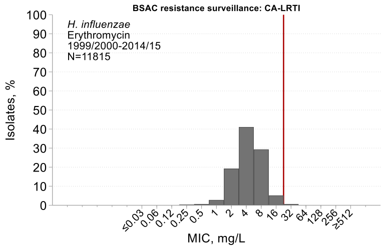

Levofloxacin

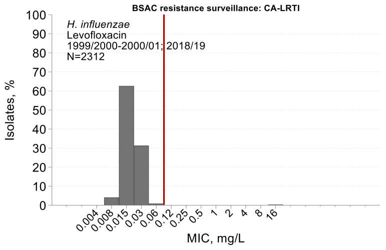

Minocycline

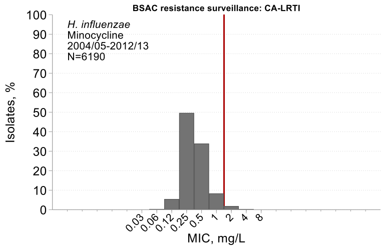

Moxifloxacin

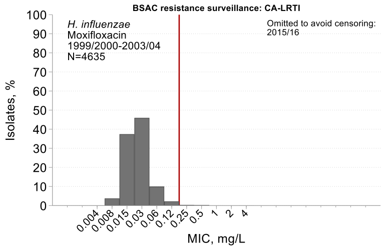

Tetracycline

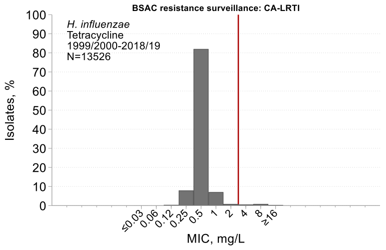

Tigecycline

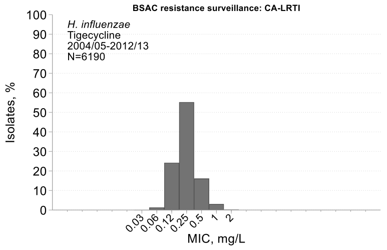

Trimethoprim

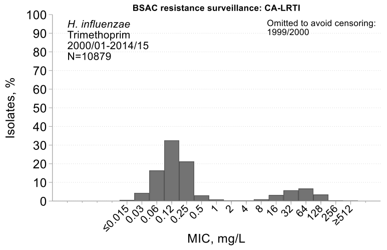

M. catarrhalis

M. catarrhalis

Amoxicillin

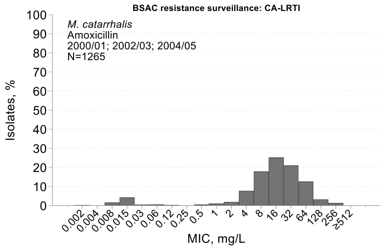

Ampicillin

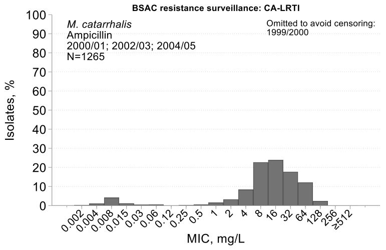

Co-amoxiclav (2:1 ratio)

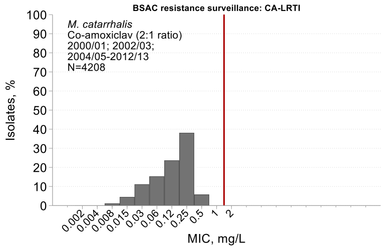

Co-amoxiclav (2 mg/L clavulanate)

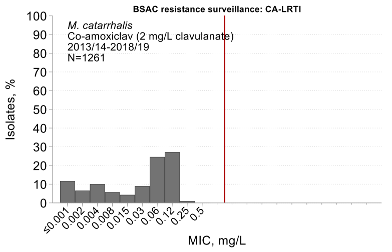

Cefaclor

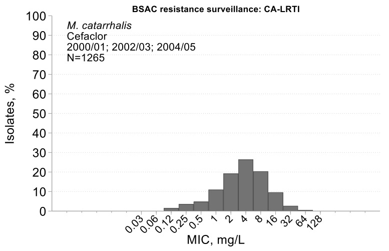

Cefotaxime

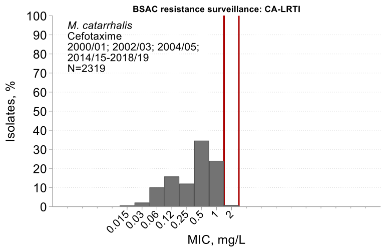

Ceftaroline - see note

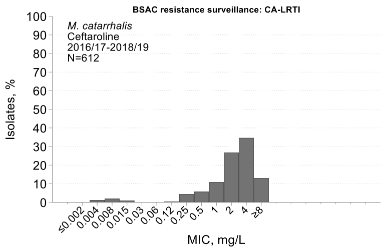

Ceftobiprole

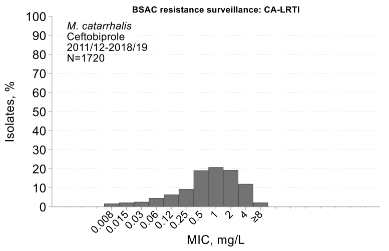

Cefuroxime

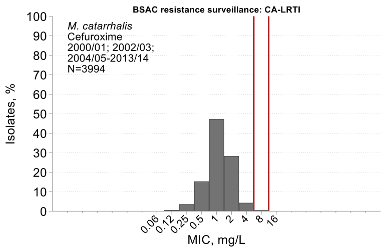

Ciprofloxacin

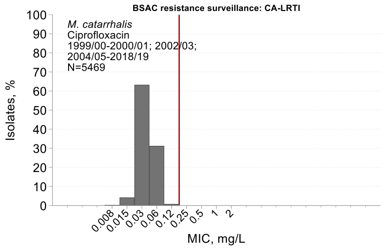

Clarithromycin

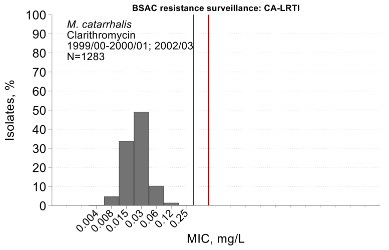

Erythromycin

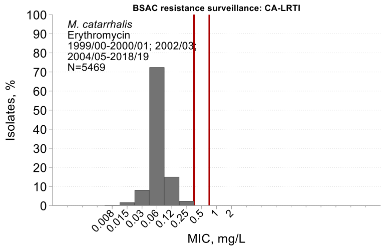

Levofloxacin

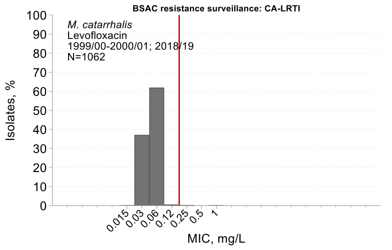

Minocycline

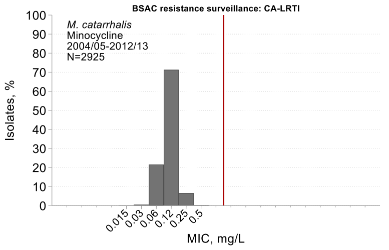

Moxifloxacin

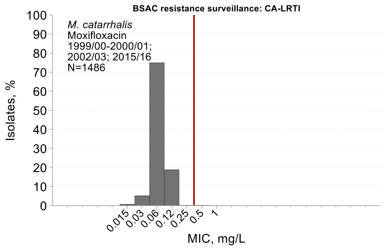

Tetracycline

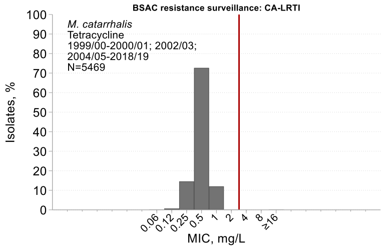

Tigecycline

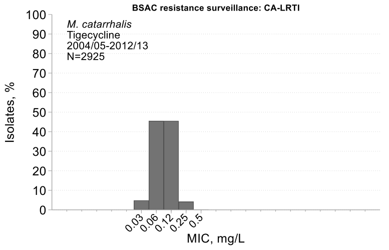

Trimethoprim

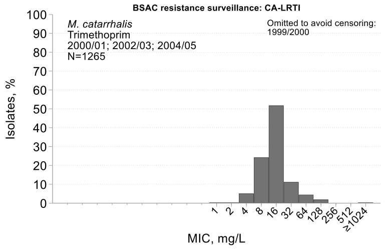

Note: approximately 13% of ceftaroline MICs for *M. catarrhalis* were censored at >4 mg/L, so plot understates the extent of higher values.
